# High-Precision and Efficient Calibration of Robot Polishing Systems Using an Adaptive Residual EKF Optimized by MIPO

**DOI:** 10.3390/s26103087

**Published:** 2026-05-13

**Authors:** Lei Wang, Yuqi Yao, Shouxin Ruan, Hainan Li, Xinming Zhang, Yiwen Zhang, Zihao Zang, Zhenglei Yu

**Affiliations:** 1 School of Mechanical and Electrical Engineering, Changchun University of Science and Technology, Changchun 130022, China; wl@mails.cust.edu.cn (L.W.); yaoyq@mails.cust.edu.cn (Y.Y.); 2024200146@mails.cust.edu.cn (Z.Z.); 2 School of Mechatronic Engineering and Automation, Foshan University, Foshan 528225, China; 3 China FAW Group Corporation Limited, Changchun 130000, China; ruanshouxin@faw.com.cn; 4 FAW Tooling Die Manufacturing Co., Ltd., Changchun 130013, China; lihainan1@faw.com.cn; 5 College of Biological and Agricultural Engineering, Jilin University, Changchun 130012, China; zlyu@jlu.edu.cn

**Keywords:** robot calibration, parrot optimization algorithm, extended Kalman filter, robot polishing system, sensor measurement

## Abstract

This paper proposes an adaptive residual extended Kalman filter method optimized by a multi-strategy improved parrot optimization algorithm (MIPO-ARKEKF) to improve the kinematic parameter calibration accuracy and efficiency of robotic polishing systems. To address the limitations of the standard extended Kalman filter (EKF), such as truncation-error accumulation during repeated linearization and sensitivity to manually selected noise parameters, an integrated improvement framework is developed. Specifically, a gradient stabilizer based on state-estimation increments is introduced to alleviate estimation degradation caused by accumulated truncation errors, while the proposed MIPO algorithm is employed to adaptively optimize the process and measurement noise covariance matrices, thereby improving the robustness of parameter identification under practical measurement uncertainty. The calibration process is established on the basis of high-precision external measurement data obtained from the robotic polishing system. In benchmark-function tests, MIPO demonstrates superior convergence performance. In physical experiments based on a KUKA KR210 R2700 robot, the proposed MIPO-ARKEKF method reduces the root mean square positioning error from 0.8927 mm to 0.4858 mm, corresponding to a 45.58% improvement in accuracy. Compared with representative hybrid calibration methods, the proposed method achieves comparable compensation accuracy while reducing computation time by 34.88% to 65.08%. Practical polishing experiments on ultra-low-expansion glass lenses further verify that the proposed method effectively improves end-effector trajectory tracking accuracy and polishing quality, providing an efficient solution for high-precision robotic polishing.

## 1. Introduction

Robots have been widely applied in optical component polishing because of their flexibility, efficiency, and cost-effectiveness, and they have become an important enabler of ultra-precision manufacturing [1,2]. As optical systems continue to evolve toward higher precision, larger apertures, and more complex surface geometries, increasingly stringent requirements are imposed on robotic polishing performance [3]. In practical polishing processes, the final surface quality is strongly affected by the absolute positioning accuracy, contact-force stability, and orientation accuracy of the robot end-effector. Although modern industrial robots can typically achieve repeatability on the order of ±0.1 mm, their absolute positioning accuracy is usually much lower than this level [4]. Moreover, manufacturing tolerances, assembly deviations, and joint wear further deteriorate the absolute positioning performance of robotic systems [5]. In precision polishing tasks, insufficient absolute positioning accuracy leads to cumulative trajectory deviations, which ultimately degrade the surface figure accuracy and integrity of optical components [6]. Therefore, improving the absolute positioning accuracy of polishing robots is a prerequisite for high-quality optical fabrication.

Existing methods for improving robot absolute positioning accuracy can generally be divided into two categories. The first is to reduce design, manufacturing, and assembly errors at the robot production stage. However, this approach depends heavily on the intrinsic precision of the robot hardware and usually involves high implementation difficulty and cost [6]. The second is error calibration and compensation, in which existing robot errors are identified and then corrected through model-based or data-driven compensation strategies [7]. Compared with hardware-level improvement, calibration is more feasible and more economical in industrial applications. The absolute positioning accuracy of robots is affected by geometric parameter errors, non-geometric errors, and measurement-related uncertainties. Among them, geometric parameter errors are generally the dominant factor and may account for more than 80% of the total positioning error [8]. Therefore, efficient and accurate calibration of geometric parameter errors remains one of the most direct and effective ways to improve the absolute positioning accuracy of polishing robots.

From a sensing perspective, robot calibration is essentially an estimation problem driven by external measurements. High-precision calibration cannot rely solely on an identification algorithm; it also depends on the sensing system used to observe robot motion. In robotic polishing, external measurement devices provide the observation basis for geometric error estimation, while their resolution, stability, workspace coverage, and noise characteristics directly influence the identification results. Therefore, improving the consistency between the calibration model and the actual sensing process is critical for obtaining reliable compensation performance in practical manufacturing scenarios.

In the field of robot geometric error calibration, many studies have focused on error identification and compensation. Nguyen L.V. et al. proposed two extended Kalman filter (EKF)-based methods that significantly improved payload attitude estimation for cable-driven parallel robots [9]. Gao G. et al. developed an adaptive unscented Kalman filter (UKF) method for kinematic parameter identification and improved the stability of robot accuracy by 25% [10]. Shen W. et al. combined particle swarm optimization (PSO) with a Kriging surrogate model to improve positioning identification accuracy [11]. Kang Z. et al. adopted a Levenberg–Marquardt and genetic algorithm hybrid (LM-GA), increasing positioning accuracy by 74.45% and alleviating parameter coupling and ill-conditioning issues [12]. Jiang G. et al. used the least absolute shrinkage and selection operator (LASSO) for kinematic error identification and significantly improved absolute positioning accuracy [13]. Chen X. et al. proposed an improved multi-objective PSO algorithm that improved the absolute positioning accuracy of a drilling robot by 26.94% [14]. Quang H.C. et al. combined EKF, double quantum-behaved PSO, and an adaptive neuro-fuzzy inference system to adaptively compute noise matrices and estimate kinematic errors, thereby improving the positioning accuracy of a five-bar parallel robot [15]. Jia H. et al. incorporated robot observability into a binary simulated annealing framework to optimize calibration data and reduce the average end-effector positioning error [16]. Liu J. et al. proposed an improved Ross’s Goose Algorithm and demonstrated its effectiveness in manipulator calibration [17]. These studies confirm the importance of combining estimation, optimization, and calibration strategies for robot kinematic error identification.

Beyond robot kinematic calibration itself, recent advances in high-precision external sensing and pose estimation have also shown increasing relevance to optical manufacturing scenarios. In particular, contemporary studies on multi-camera relative pose estimation, generalized pose recovery, and affine-correspondence-based geometric reconstruction indicate that external measurement systems are evolving from single-sensor observation toward richer multi-sensor and vision-assisted configurations [18]. Although these approaches mainly address external pose recovery rather than robot geometric parameter calibration, they provide useful insight into how future calibration systems may achieve broader workspace coverage, stronger sensing flexibility, and improved adaptability in complex optical-manufacturing environments [19,20]. From this perspective, the sensor-guided calibration framework developed in this work is not limited to the present laser-tracker-based implementation, but may also be extended to future vision-assisted or multi-sensor robotic polishing scenarios. In contrast to these sensing-oriented pose-estimation approaches, the present study focuses on the calibration side of the problem, namely, how external measurements can be incorporated into robot geometric error identification and compensation in an efficient and robust manner.

Nevertheless, several limitations remain in existing methods. The Levenberg–Marquardt algorithm handles nonlinear problems through first-order Taylor approximation, which inevitably introduces truncation errors and may lead to cumulative estimation bias [21]. UKF can provide better nonlinear estimation performance, but it usually incurs a relatively high computational burden because of sigma-point propagation. In addition, its performance is sensitive to the setting of the process and measurement noise covariance matrices [22]. EKF can often achieve performance close to UKF with lower computational cost, but it is still sensitive to initialization and may also suffer from approximation degradation caused by repeated linearization [23]. Metaheuristic algorithms such as PSO and GA have strong global-search capability, but they may prematurely converge or become trapped in local optima when solving nonlinear and coupled optimization problems [2]. Although hybrid methods can improve identification accuracy, they often increase implementation complexity and computational cost.

This study focuses on low-speed and low-force precision polishing scenarios, in which geometric errors are the dominant source of positioning inaccuracy. To address the above issues, this paper proposes a sensor-guided calibration framework for robotic polishing systems based on a multi-strategy improved parrot optimization algorithm and an adaptive residual extended Kalman filter (MIPO-ARKEKF). In this framework, external high-precision measurements are treated as the observation basis for geometric error estimation, and the sensing uncertainty is incorporated into the adaptive optimization of the filtering process. Specifically, an increment-based residual stabilization mechanism is introduced into the EKF to mitigate the degradation caused by accumulated truncation errors during repeated linearization. Meanwhile, the proposed MIPO algorithm is used to adaptively optimize the process and measurement noise covariance matrices, thereby reducing the dependence on manually selected initial parameters and improving robustness under sensor-dependent uncertainty.

The choice of parrot optimization as the optimization backbone is motivated not only by its benchmark performance but also by its search mechanism. The covariance-optimization problem considered in robot calibration is continuous, nonlinear, and moderately coupled, requiring both sufficient global exploration and stable local exploitation. Compared with directly using a conventional metaheuristic as a generic tuning tool, the PO framework provides a suitable structural basis for balancing these two requirements. Based on this foundation, the proposed MIPO enhances the original PO algorithm through Chebyshev chaotic initialization, adaptive weighting, and dynamic information sharing, thereby improving population diversity, convergence stability, and the ability to escape local optima.

The main contributions of this work are as follows. First, a sensor-guided high-precision calibration framework for robotic polishing systems is established, in which external measurement data are explicitly integrated into the geometric error estimation process. Second, a residual stabilization mechanism based on state-estimation increments (GSBISE) is introduced to improve EKF robustness against truncation-error accumulation. Third, the proposed MIPO algorithm adaptively optimizes the process and measurement noise covariance matrices, improving estimation robustness under sensing uncertainty while reducing dependence on manually selected initial values. Finally, the effectiveness of the overall framework is validated through CEC 2022 benchmark tests, robot calibration simulations, and practical polishing experiments on ultra-low-expansion glass.

The remainder of this paper is organized as follows. Section 2 introduces the robot polishing system, the external perception architecture, as well as the corresponding kinematics and error models. It also presents the proposed error identification method based on MIPO-ARKEKF. Section 3 reports the simulation and experimental results. Section 4 concludes the paper and discusses future work.

## 2. Materials and Methods

### 2.1. The Kinematics and Error Model of the Robot Polishing System

#### 2.1.1. Robot Polishing System and External Sensing Architecture

**Figure 1:**
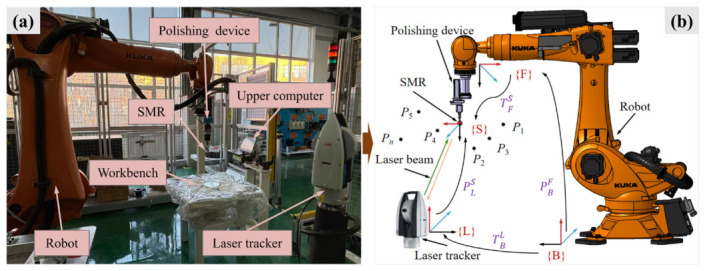
Description of the experimental system. (**a**) Robot polishing system and measurement device; (**b**) robot coordinate system.

Figure 1a provides a detailed description of the robotic polishing system and the measurement equipment. The robot model used in this study is the KUKA KR 210 R2700, which performs polishing tasks by controlling the polishing tool attached to its end-effector. Low absolute positioning accuracy of the robot can adversely affect its tracking precision of dwell points along the polishing path, thereby compromising the machining quality of the optical component. The measurement setup consists of a laser tracker, a spherically mounted retroreflector (SMR), and a host computer. The Leica AT960 laser tracker, used for high-precision spatial measurements, serves as the core external sensing layer in the proposed calibration framework. The Leica AT960 laser tracker provides a measurement accuracy of up to ±10 µm + 6 µm/m (2*σ*), providing sub-40 μm precision across the typical 2–5 m working distances of our robotic polishing cell. This setup enables accurate geometric error estimation of the robot’s end-effector. The SMR is mounted on the robot end-effector to reflect laser beams and facilitate precise spatial measurements. The host computer is responsible for data acquisition, coordinate transformation, and calibration-data management. Figure 1b illustrates the coordinate systems established based on the configuration shown in Figure 1a.

In Figure 1b, {B} represents the robot’s base coordinate system. {F} denotes the flange coordinate system at the robot’s end-effector. {S} is the coordinate system of the SMR. {L} corresponds to the laser tracker’s coordinate system. The robot positions the SMR at various points *P_i_* (*i* = 1, 2, …, N) in space, measuring both the theoretical and actual positions of each point. The positioning error at point Pi can be expressed by Formula (1).

(1) $$∆P_{i}=P_{i}-P_{i}^{\prime }$$

where *P_i_* = [*x_i_*, *y_i_*, *z_i_*] represents the actual position of the *i*-th point, and *P_i_*’ = [*x_i_*’, *y_i_*’, *z_i_*’] represents the theoretical position of the *i*-th point.

The specific expressions for *P_i_* and *P_i_’* are given in Equation (2).

(2) $$\left\{\begin{array}{c} P_{i}=P_{L}^{S}\left(i\right)\cdot T_{B}^{L}\\ P_{i}^{\prime }=P_{B}^{F}\left(e,Q_{i}\right)\cdot T_{F}^{S} \end{array}\right.$$

where $P_{B}^{F}(e,Q_{i})$ denotes the transformation matrix from coordinate system {F} to coordinate system {B}. Here, the robot’s geometric error parameters are precisely the errors that require compensation. *Q_i_* is the joint angle vector at the *i*-th point. $T_{B}^{L}$ represents the transformation matrix from coordinate system {B} to coordinate system {L}, which can be directly computed via the measurement software. $T_{F}^{S}$ denotes the transformation matrix from coordinate system {F} to coordinate system {S}, which can be calculated based on the robot′s structure. $P_{L}^{S}(i)$ are the coordinates of the *i*-th point in coordinate system {L}, measured by the laser tracker. Therefore, to achieve the desired absolute positioning accuracy of the robot, it is necessary to compensate for the error parameters *e*.

#### 2.1.2. Kinematic and Error Model

**Figure 2:**
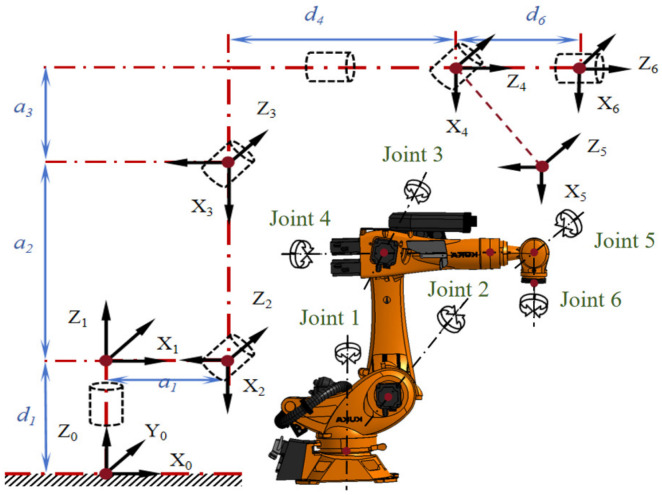
Diagram of the KUKA KR 210 R2700 robot coordinate system.

Figure 2 illustrates the coordinate system of the KUKA KR 210 R2700 robot, established using the Modified Denavit–Hartenberg (MD-H) method [24]. Table 1 presents the geometric parameters of the KUKA KR 210 R2700 robot, which constitute the geometric parameter vector *e* ∈ *R*^24×1^.

In the MD-H model, the homogeneous transformation from coordinate system {*x*_*i*−1_, *y*_*i*−1_, *z*_*i*−1_} to coordinate system {*x_i_*, *y_i_*, *z_i_*} is described by Equation (3).

(3) Tii−1=Transxi−1,ai−1·Rotxi−1,αi−1·Transzi,di·Rotzi,θi     =cosθi−sinθi0ai−1sinθicosαi−1cosθicosαi−1−sinαi−1−disinαi−1sinθisinαi−1cosθisinαi−1cosαi−1dicosαi−10001

In the equation, *Rot* (·) represents a rotation transformation, and *Trans* (·) denotes a translation transformation; *a*_*i*−1_ is the length of link *i*; *d_i_* is the offset distance of link *i* relative to link *i* − 1; $\alpha_{i-1}$ denotes the twist angle of link *i* relative to link *i* − 1; and *θ_i_* is the joint angle of link *i* relative to link *i* – 1.

The pose transformation matrix ^0^*T*_6_ of the end of the robotic arm relative to the base coordinate system is shown in Equation (4).

(4) T60=T10·T21·T32·T43·T54·T65

Therefore, the position *P_r_* of the robot’s end-effector can be represented by its end coordinates (*x*_6_, *y*_6_, *z*_6_), which can be mapped to the coordinates *(x*_0_, *y*_0_, *z*_0_*)* in the base coordinate system through kinematic transformation.

(5) Pre,Q=PrxPryPrz=T601,4T602,4T603,4=PBFe,Q

The geometric parameter error vector of the system is shown in Equation (6).

(6) $$∆e={\left[∆\alpha_{1}\cdots ∆\alpha_{6}\;∆a_{1}\cdots ∆a_{6}\;∆d_{1}\cdots ∆d_{6}\;∆\theta_{1}\cdots ∆\theta_{6}\right]}^{T}$$

Taking into account the geometric parameter deviations, a motion error model for the robot can be constructed. The calculation expression of this model is shown in Equation (7).

(7) T60+∆T60=∏i=16(Tii−1+∆Tii−1)

The difference form of Formula (7) can be expressed as Formula (8).

(8) $${∆}^{0}T_{6}=\sum_{i=1}^{6}\left(\frac{\partial^{i-1}T_{i}}{\partial a_{i-1}}∆a_{i-1}+\frac{\partial^{i-1}T_{i}}{\partial d_{i}}∆d_{i}+\frac{\partial^{i-1}T_{i}}{\partial \alpha_{i-1}}+\frac{\partial^{i-1}T_{i}}{\partial \theta_{i}}∆\theta_{i}\right)$$

According to Equation (8), its matrix form can be expressed as Formula (9).

(9) $$∆P_{r}=J\cdot ∆e$$

where Δ*P_r_* represents the positioning error of the robot, *J* is a Jacobian matrix that establishes the mapping relationship from geometric parameter errors to end positioning errors, and Δ*e* is the geometric parameter error that needs to be identified.

In particular, the extended form of Equation (9) is:

(10) $$\left[\begin{array}{c} ∆P_{r}x\\ ∆P_{r}y\\ ∆P_{r}z \end{array}\right]=J\cdot ∆e=\left|\begin{array}{cccc} \frac{\partial P_{r}x}{\partial \alpha } & \frac{\partial P_{r}x}{\partial a} & \frac{\partial P_{r}x}{\partial \theta } & \frac{\partial P_{r}x}{\partial d}\\ \frac{\partial P_{r}y}{\partial \alpha } & \frac{\partial P_{r}y}{\partial a} & \frac{\partial P_{r}y}{\partial \theta } & \frac{\partial P_{r}y}{\partial d}\\ \frac{\partial P_{r}z}{\partial \alpha } & \frac{\partial P_{r}z}{\partial a} & \frac{\partial P_{r}z}{\partial \theta } & \frac{\partial P_{r}z}{\partial d} \end{array}\right|\cdot \left[\begin{array}{c} ∆\alpha \\ ∆a\\ ∆\theta \\ ∆d \end{array}\right]$$

Therefore, the motion error model of this robot polishing system takes the form shown in Equation (11).

(11) $$∆P_{i}=P_{L}^{S}\left(i\right)\cdot T_{B}^{L}-P_{r}\left(e,Q_{i}\right)\cdot T_{F}^{S}=J_{i}\cdot ∆e\cdot T_{F}^{S}$$

In conclusion, this section establishes the kinematic model and error model of the robot polishing system. The kinematic model is expressed by Formula (7), and the error model is expressed by Formula (11).

### 2.2. Robot Polishing System Calibration Based on MIPO-ARKEKF

**Figure 3:**
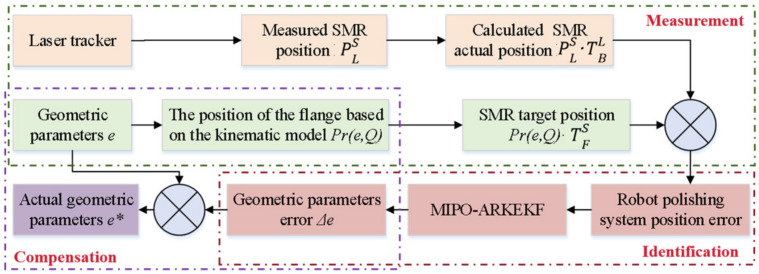
The calibration process of the robot polishing system MIPO-ARKEKF.

As illustrated in Figure 3, the calibration procedure for the robotic polishing system operates as follows. Based upon the kinematic model and error model of the robot, the system controls the robot to navigate to arbitrary positions within the workspace. A laser tracker is employed to measure the position *P_n_* of the robotic polishing tool. Through iterative measurements, the theoretical coordinates and actual coordinates of n spatial positions are acquired. Subsequently, the MIPO-ARKEKF algorithm is utilized to identify the geometric parameter errors Δ*e* of the robot. The identified error parameters Δ*e* are then uploaded to the robot controller. During actual polishing operations, the controller employs the compensated error model for trajectory planning and inverse kinematics computation, thereby enabling real-time correction of the end-effector command positions to achieve accuracy enhancement.

#### 2.2.1. Geometric Error Identification Based on Extended Kalman Filter

The EKF algorithm is widely utilized for error identification. Its fundamental principle involves the linearization of nonlinear systems based on a first-order Taylor series expansion. By performing the Taylor series expansion of the nonlinear functions *f*(·) and *h*(·) around the estimated value $\hat{x}$*_k_* the linearized Equations (12) and (13) are derived [25].

(12) $$f\left(x_{k}\right)=f\left({\hat{x}}_{k\left|k\right.}\right)+F_{k}\left(x_{k}-{\hat{x}}_{k\left|k\right.}\right)+o\left(x_{k}-{\hat{x}}_{k\left|k\right.}\right)$$

(13) $$h\left(x_{k}\right)=h\left({\hat{x}}_{k\left|k-1\right.}\right)+H_{k}\left(x_{k\left|k-1\right.}\right)+o\left(x_{k}-{\hat{x}}_{k\left|k-1\right.}\right)$$

In the equation, $F_{k}={\frac{\partial f}{\partial x}|}_{x={\hat{x}}_{k|k}}$, $H_{k}={\frac{\partial h}{\partial x}|}_{x={\hat{x}}_{k|k-1}}$ represent the Jacobian matrices of the state function f and the measurement function *h*, respectively. In this study, the robot error model establishes the relationship between the end-effector position error ∆*P_i_* and the geometric parameter errors ∆*e* by utilizing the sensitivity of ∆*P_i_* to ∆*e*, which is characterized by the error matrix *J_i_*. Consequently, *J_i_* corresponds to the measurement matrix *H_k_* in this formulation.

$o(x_{k}-{\hat{x}}_{k|k})$ and $o(x_{k}-{\hat{x}}_{k|k-1})$ represent the expressions for the higher-order terms in the Taylor series expansion. By neglecting these higher-order terms, the system’s state equation and measurement equation can be derived as shown in Equations (14) and (15).

(14) $$x_{k}=f\left(x_{k-1},u_{k-1}\right)+\omega_{k}$$

(15) $$z_{k}=h\left(x_{k}\right)+v_{k}$$

where *ω_k_* denotes the process noise and *v_k_* denotes the measurement noise at the *k*-th iteration. Both are assumed to be zero-mean Gaussian noise sequences with covariance matrices *S_k_* and *R_k_*, respectively.

Since the robot’s geometric errors are static parameters, the state equation can be simplified to Equation (16).

(16) $${\hat{x}}_{k\left|k-1\right.}={\hat{x}}_{k-1\left|k-1\right.}$$

where ${\hat{x}}_{k|k-1}$ represents the predicted state at the k-th iteration, ${\hat{x}}_{k-1|k-1}$ denotes the actual value from the (*k* − 1)-th iteration, and $x\in R^{24\times 1}$ corresponds to the geometric parameter error, which is defined as Δ*e* in Equation (11).

The covariance matrix *C* ∈ *R*^24×24^ is predicted using Formula (17).

(17) $$C_{k\left|k-1\right.}=F_{k-1}C_{k-1\left|k-1\right.}F_{k-1}^{T}+S_{k-1}$$

Similarly, after simplifying the state equation, *F_k−1_* = *I* (the identity matrix), so the covariance matrix prediction can be simplified to Formula (18).

(18) $$C_{k\left|k-1\right.}=C_{k-1\left|k-1\right.}+S_{k-1}$$

where *C_k|k_*_−1_ represents the predicted covariance for the *k*-th iteration, while *C*_*k*−1|*k*−1_ represents the actual covariance for the (*k* − 1)-th iteration. *S* ∈ *R*^24×24^ is the system noise covariance matrix for the (k − 1)-th iteration.

The observed values of position errors are calculated using Formula (19).

(19) $$z_{k}=H_{k}{\hat{x}}_{k}+E_{k}$$

where *z_k_* ∈ *R*^3*n*×1^ represents the positional error, *H_k_* ∈ *R*^3*n*×24^ is the Jacobian matrix of the error, and *E_k_* ∈ *R*^3*n*×1^ is the observation error.

The Kalman gain *K_k_* ∈ *R*^24×3*n*^ is updated through Formula (20).

(20) $$K_{k}=C_{k\left|k-1\right.}H_{k}^{T}{\left(H_{k}C_{k\left|k-1\right.}H_{k}^{T}+R_{k}\right)}^{-1}$$

where *R_k_* ∈ *R*^3*n*×3*n*^ represents the covariance matrix of the measurement noise for *k* iterations.

The parameter status update is calculated using Formula (21).

(21) $${\hat{x}}_{k\left|k\right.}={\hat{x}}_{k\left|k-1\right.}+K_{k}\left(z_{k}-H_{k}{\hat{x}}_{k\left|k-1\right.}\right)$$

where ${\hat{x}}_{k|k}$ represents the posterior estimate after *k* iterations.

The covariance is updated using Formula (22).

(22) $$C_{k\left|k\right.}=\left(I-K_{k}H_{k}\right)C_{k\left|k-1\right.}$$

where *I* ∈ *R*^24×24^ represents the identity matrix, and *C*_*k*|*k*_ represents the posterior covariance after *k* iterations.

Therefore, the EKF algorithm, together with the established kinematic model of the robot and the error model, can be used to identify the geometric parameter error ${\hat{x}}_{k}$. By compensating for the identified error, the actual parameter error can be obtained through Formula (23).

(23) $$e*=e+∆e=e+\hat{x}$$

#### 2.2.2. Gradient Stabilizer Based on the Increment in State Estimation

The EKF algorithm achieves local linearization of nonlinear systems through a first-order Taylor series expansion. During this linearization process, higher-order terms are directly truncated. As the number of iterations increases, the error resulting from the cumulative effect of these neglected higher-order terms becomes progressively larger. This accumulation of error gradually diminishes or invalidates the “gradient” information that the algorithm uses to guide parameter updates, ultimately compromising estimation accuracy. To address this limitation, a gradient stabilizer based on state estimation increments is introduced (GSBISE). This approach is inspired by the methodology used in residual neural networks to counteract network degradation, a technique that has been successfully applied and validated within nonlinear systems [26].

The definition of this gradient stabilizer is given by Formula (24).

(24) $$∆{\hat{x}}_{k\left|k\right.}={\hat{x}}_{k\left|k\right.}-{\hat{x}}_{k-1\left|k-1\right.}$$

where $∆{\hat{x}}_{k|k}$ represents the state estimation increment after *k* iterations. This increment is used to compensate the initial value for the next iteration. Therefore, the initial value for the (*k*+1)-th iteration is expressed by Formula (25).

(25) $${\hat{x}}_{k\left|k\right.}={\hat{x}}_{k\left|k\right.}+∆{\hat{x}}_{k\left|k\right.}$$

#### 2.2.3. Adaptive Optimization of EKF Noise Covariance Matrices Using MIPO

In the standard EKF, the noise matrices C, S, and R are predefined fixed values that require additional manual specification prior to identification. Owing to their static nature, these matrices fail to reflect the authentic noise characteristics, thereby directly compromising the estimation accuracy of the algorithm. Furthermore, the assignment of these values exhibits substantial dependency upon a priori knowledge, with no guarantee of achieving optimal numerical configurations. To address this limitation, this study introduces metaheuristic algorithms for adaptive optimization of these matrices. Given that the PO algorithm has demonstrated favorable global search capabilities in prior investigations, we have enhanced this algorithm to execute the aforementioned optimization task. We initially incorporate an adaptive factor to dynamically adjust and identify optimal noise matrices, thereby achieving superior identification performance. Subsequently, the optimal solution for the adaptive factor is obtained through the MIPO algorithm. Following the introduction of the adaptive factor, matrices *C*, *S*, and *R* can be expressed by Equation (26).

(26) $$\left\{\begin{array}{c} C=\alpha \cdot C_{0};\\ S=\beta \cdot S_{0};\\ R=\gamma \cdot R_{0} \end{array}\right.\;$$

where *C*_0_, *S*_0_, and *R*_0_ represent the zero-mean Gaussian white noise covariance matrices initially selected from the robotic polishing system. The parameters *α*, *β*, and *γ* are introduced as adaptive factors. By adjusting the magnitude of these parameters, the optimal values for *C*, *S*, and *R* that maximize the identification accuracy of the EKF can be determined. Furthermore, based on the specific characteristics of the robot error identification process, the values of *α*, *β*, and *γ* are constrained to specific ranges. Specifically, *α* ∈ [10^−7^, 10^−5^], *β* ∈ [10^−7^, 10^−5^], and *γ* ∈ [10^−5^, 10^−3^], which are collectively represented by the vector *ω*.

(27) $$w=\left(\alpha ,\beta ,\gamma \right)$$

The values of *C*_0_, *S*_0_, and *R*_0_ are initialized through Formula (28).

(28) $$\left\{\begin{array}{c} C_{0}=\mathrm{diag}\left(randn\left(24,1\right)\right);\\ S_{0}=\mathrm{diag}\left(randn\left(24,1\right)\right);\\ R_{0}=\mathrm{diag}\left(randn\left(3n,1\right)\right). \end{array}\right.$$

where randn() denotes a random number (or vector) drawn from a standard normal distribution, 24 represents the 24 parameter errors accumulated from the identification errors, *n* represents the sample size, and 3 indicates that the three directions of the coordinate axes are *x*, *y*, and *z*.

In robotic error identification systems, the EKF accurately estimates actual parameter errors through the identification of measurement data, thereby reducing the overall system error. This process employs the MIPO algorithm to achieve dynamic optimization of the adaptive factors. Given that the identification objective is to enhance the positioning accuracy of the polishing robot, the robot’s positioning error is selected as the fitness function to evaluate the accuracy of the adaptive factors. The specific formulation of the fitness function is provided in Equation (29).

(29) $$\left\{\begin{array}{c} f_{obj}\left(C,S,R\right)=F\left({\hat{x}}_{EKF}\right)\\ f=\frac{1}{n}\sum_{i=1}^{n}\sqrt{{\left|∆P_{i}\right|}^{2}}=\frac{1}{n}\sum_{i=1}^{n}\sqrt{{\left|J_{i}{\hat{x}}_{EKF}\left(C,S,R\right)\cdot T_{F}^{s}\right|}^{2}} \end{array}\right.$$

where f represents the error function for robot calibration; *n* denotes the number of samples; and *x_EKF_* indicates the error in the motion parameters obtained through EKF identification estimation.

### 2.3. Parrot Optimization Algorithm Based on Multi-Strategy Improvement (MIPO)

#### 2.3.1. Review of PO

**Figure 4:**
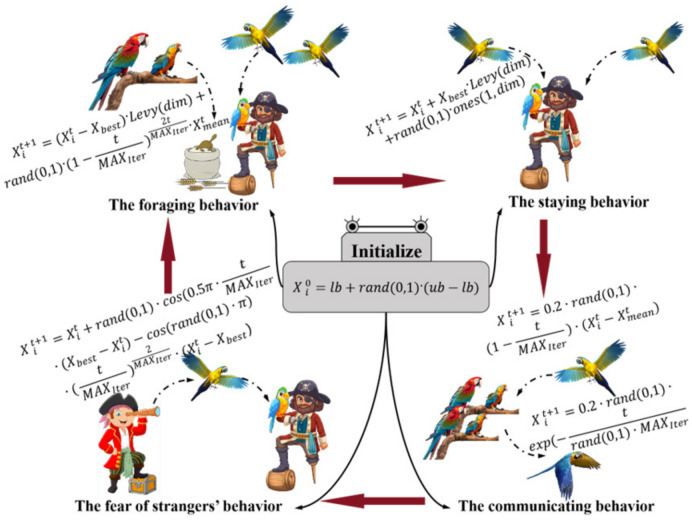
Four behaviors of the parrot optimization algorithm.

The PO is a high-efficiency search strategy inspired by the ethological repertoire of trained Pyrrhura molinae parrots; its conceptual framework is synthesized into four principal behaviors: foraging, resting, vocal communication, and neophobia toward unfamiliar conspecifics [27]. By mathematically emulating these natural behaviors, the algorithm iteratively refines the positions of individual agents to converge upon an optimal locus. Specifically, the foraging behavior directs an agent toward the globally best solution and the spatial centroid of the population, thereby intensifying exploitation. The resting behavior introduces a localized random perturbation around the incumbent best, facilitating fine-grained search. The communication behavior models information exchange within the flock: with equal probability an agent either moves toward the population mean to reinforce social cohesion or executes an exploratory leap to maintain diversity. Finally, the fear-of-strangers behavior implements an avoidance mechanism that repels agents from underperforming regions, thus mitigating premature convergence to sub-optimal attractors. These four behavioral primitives are illustrated schematically in Figure 4.

Let the parrot population be of size N, the feasible decision space be delimited by the lower bound vector *lb* and the upper bound vector *ub*, and the maximum number of iterations be *MAX_Iter_*. The stochastic initialization of the population is then formally expressed by Equation (30).

(30) $$X_{i}^{0}=lb+rand\left(0,1\right)\cdot \left(ub-lb\right)$$

where *rand* (0, 1) denotes a scalar drawn from the uniform distribution on the interval [0, 1], and $X_{i}^{0}$ is the initial position of the *i*-th individual.

After the population initialization is completed, each parrot updates its spatial position based on the four behavioral characteristics of the population. This gradually achieves the goal of seeking the optimal solution.

During the foraging behavior stage, the parrots determine the approximate location of the food by observing it or considering the position of the owner. After making the judgment, they fly to the corresponding location. The position update is as shown in Formula (31).

(31) $$X_{i}^{t+1}=\left(X_{i}^{t}-X_{best}\right)\cdot Levy\left(dim\right)+rand\left(0,1\right)\cdot {\left(1-\frac{t}{MAX_{Iter}}\right)}^{\frac{2t}{MAX_{Iter}}}\cdot X_{mean}^{t}$$

where *t* represents the current iteration number, and $X_{i}^{t}$ and $X_{i}^{t+1}$ denote the position of the *i*-th parrot at iteration numbers *t* and *t* + 1, respectively. *X_best_* represents the optimal solution from initialization to the current search, and also represents the current position of the owner. *Levy(dim)* represents the Levy distribution of the parrot’s flight. ($X_{i}^{t\;}$− X_best_) indicates the movement of the parrot relative to the owner’s position. $X_{imean}^{t}$ represents the average position of all parrots. The average position is expressed by Formula (32).

(32) $$X_{mean}^{t}=\frac{1}{N}\sum_{k=1}^{N}X_{k}^{t}$$

The Levy distribution can be obtained using Formula (33), with the value of *δ* set to 1.5, and *Γ*(⋅) denotes the Gamma function.

(33) $$\left\{\begin{array}{c} Levy\left(dim\right)=\frac{\mu \cdot \sigma }{{\left|v\right|}^{\frac{i}{\delta }}}\\ \mu ∼N\left(0,dim\right)\\ v∼N\left(0,dim\right)\\ \sigma =\left(\frac{\Gamma \left(1+\delta \right)\cdot \sin\left(\frac{\pi \delta }{2}\right)}{\Gamma \left(\frac{1+\delta }{2}\right)\cdot \delta \cdot 2^{\frac{1+\delta }{2}}}\right) \end{array}\right.$$

During the stay behavior stage, the parrot suddenly flies to any part of the owner’s body and stays there for a certain period of time. The position update is as per Formula (34).

(34) $$X_{i}^{t+1}=X_{i}^{t}+X_{best}\cdot Levy\left(dim\right)+rand\left(0,1\right)\cdot ones\left(1,dim\right)$$

where *Rand* (0, 1) · *Ones(1, dim)* represents the process of the parrot randomly staying on the owner. *Ones(1*, *dim)* represents a vector consisting entirely of 1 s in the dimension dim. *Xbest*·*Levy(dim)* represents the flight of the parrot towards the owner.

In natural environments, parrots are social animals, a trait that persists even in trained individuals. They engage in information exchange through vocalizations. This communicative behavior presents two options: returning to the flock or not returning after interaction. It is assumed that both behaviors occur with equal probability, and the current average position of the population is used to represent the center of the parrot flock. During this phase, a randomly generated probability value *P* within the range [0, 1] determines the specific behavior. When *p* ≤ 0.5, the parrot joins the flock; conversely, if *p* > 0.5, it flies away immediately after communication. The position update rule corresponding to this behavior is given by Equation (35).

(35) $$X_{i}^{t+1}=\left\{\begin{array}{c} 0.2\cdot rand\left(0,1\right)\cdot \left(1-\frac{t}{MAX_{Iter}}\right)\cdot \left(X_{i}^{t}-X_{mean}^{t}\right),\;P\leq 0.5\\ 0.2\cdot rand\left(0,1\right)\cdot \exp\left(-\frac{t}{rand\left(0,1\right)\cdot MAX_{Iter}}\right),\;P>0.5 \end{array}\right.$$

When a parrot encounters a stranger, its survival instinct drives it to maintain distance from humans and seek a safer environment. To avoid potential conflicts or other dangers posed by the unfamiliar individual, the parrot employs specific avoidance strategies. The corresponding position update is described by Equation (36).

(36) $$X_{i}^{t+1}=X_{i}^{t}+rand\left(0,1\right)\cdot \cos\left(0.5\pi \cdot \frac{t}{MAX_{Iter}}\right)\cdot \left(X_{best}-X_{i}^{t}\right)\;\;\;-\cos\left(rand\left(0,1\right)\cdot \pi \right)\cdot {\left(\frac{t}{MAX_{Iter}}\right)}^{\frac{2}{MAX_{Iter}}}\cdot \left(X_{i}^{t}-X_{best}\right)$$

The component $cos(rand(0,1)\cdot \pi )\cdot {\left(\frac{t}{{MAX}_{Iter}}\right)}^{\frac{2}{{MAX}_{Iter}}}\cdot (X_{i}^{t}-X_{best})$ models the behavior of a parrot maintaining distance from strangers. The other component, $rand\cdot (0,1)\cdot cos(0.5\cdot \frac{t}{{MAX}_{Iter}})\cdot (X_{best}-X_{i}^{t})$, represents the parrot’s behavior when evading the owner or approaching the optimal position.

#### 2.3.2. Improving the PO Algorithm Through Multiple Strategies

(1) Chebyshev chaotic mapping initialization strategy

The PO algorithm typically employs stochastic methods to generate the initial population. This simplistic random distribution mechanism may lead to non-uniform coverage of the search space by the population individuals. Consequently, certain regions might be over-represented while others remain insufficiently explored, ultimately constraining population diversity [28]. This deficiency in initial population diversity can predispose the algorithm to converge towards local optima prematurely, rather than facilitating an effective search towards the global optimum. Particularly when addressing complex nonlinear optimization problems, this limitation systematically undermines the algorithm’s global exploration capability.

Recognizing that the quality of the initial solution is critically linked to the algorithm’s ultimate convergence speed and precision, deficiencies in the initial population distribution can lead to slower global convergence and reduced search efficiency. To address this issue, a chaotic mapping strategy is introduced into the parrot optimization (PO) algorithm for population initialization. Chaotic mapping generates sequences characterized by high ergodicity and unpredictability, enabling a more uniform and widespread distribution of the initial population within the solution space [29]. This approach establishes a superior foundation for subsequent phases of global exploration and local exploitation, thereby enhancing the overall optimization performance.

The Chebyshev map is characterized by its desirable properties of ergodicity and uniformity, which contribute to a comprehensive exploration of the search space. Additionally, its computational complexity remains relatively low, ensuring rapid execution during the initialization phase and thereby maintaining overall algorithm efficiency [30]. These attributes make the Chebyshev map particularly well suited for addressing the high-precision optimization demands inherent in the PO algorithm.

The mathematical expression of the Chebyshev mapping is as shown in Formula (37).

(37) $$x_{k+1}=\cos\left[\tau \cdot \arccos\left(x_{k}\right)\right]$$

where *x_k_* ∈ [−1, 1] represents the value at the *k*-th iteration. The constant *τ* denotes the degree of the polynomial, which governs the chaotic behavior of the system; in this paper, *τ* = 2. When *τ* = 1, the map exhibits an identity transformation with no chaotic behavior; for τ ≥ 2 it demonstrates chaotic dynamics, and for *τ* ≥ 3, it becomes a strongly chaotic map, indicating that the degree of chaos intensifies with increasing *τ*. Furthermore, the map exhibits symmetry about the origin when *τ* is an even number, while no such symmetry is observed when *τ* is odd.

The individual *x_k_* generated by the Chebyshev method will be mapped to the actual search space [*lb*, *ub*], which can be achieved with Formula (38).

(38) $$X_{i}^{0}=lb+\frac{x_{k}+1}{2}\cdot \left(ub-lb\right)$$

(2) Weight adaptive adjustment mechanism

During the communication phase of the PO algorithm, information transfer between individuals is highly stochastic. A random number *H*, uniformly distributed within [0, 1], determines whether an individual joins the population. While this mechanism is straightforward to implement, its inherent uncertainty compromises the search capability of the PO algorithm during the initial stages, significantly impacting global exploration. To address this limitation, this study establishes a weight adaptive adjustment mechanism aimed at enhancing the dynamic balance between global exploration and local exploitation within the communication behavior. Aligned with the characteristics of the PO algorithm and intending to emphasize global exploration in the early phase and local exploitation in the later phase, two adaptive weight factors, *ω*_1_ and *ω*_2_, are introduced. These factors operate on the exploration and exploitation stages, respectively, and adapt dynamically as the number of iterations increases. The specific formulation is provided in Equation (39).

(39) $$\left\{\begin{array}{c} \omega_{1}=0.9\cdot e^{-3\cdot {\left(\frac{t}{MAX_{Iter}}\right)}^{2}}\\ \omega_{2}=0.1+0.8\left(1-e^{-3\cdot {\left(\frac{t}{MAX_{Iter}}\right)}^{2}}\right) \end{array}\right.$$

where *t* represents the current iteration number, and *MAX_Iter_* is the maximum number of iterations.

To address the limitations of sequential phases in the PO algorithm, this study effectively integrates global exploration and local exploitation through adaptive factors, as specifically formulated in Equation (40). During the communication phase, a larger weight *ω*_1_ in the early search stage guides the parrots to prioritize external influences over internal communication, thereby enhancing population diversity and global exploration capability. Conversely, a larger weight *ω*_2_ in the later search stage directs the parrots to focus more on internal communication, which improves the local exploitation capability.

(40) $$X_{i}^{t+1}=0.9\cdot e^{-3\cdot {\left(\frac{t}{MAX_{Iter}}\right)}^{2}}\cdot 0.2\cdot rand\left(0,1\right)\cdot \left(1-\frac{t}{MAX_{Iter}}\right)\cdot \exp\left(-\frac{t}{rand\left(0,1\right)\cdot MAX_{Iter}}\right)\;+\left[\left(0.1+0.8\cdot \left(1-e^{-3\cdot {\left(\frac{t}{MAX_{Iter}}\right)}^{2}}\right)\right)\cdot 0.2\cdot rand\left(0,1\right)\cdot \left(1-\frac{t}{MAX_{Iter}}\right)\cdot \left(X_{i}^{t}-X_{mean}^{t}\right)\right]$$

(3) Dynamic Information Sharing Mechanism

Similar to many other optimization algorithms, the PO algorithm tends to fall into local optima when handling complex tasks. During the later iterations of the PO algorithm, the diversity of the parrot population significantly decreases, causing individuals to cluster around the perceived optimal solution. If this optimal solution is trapped in a local optimum, the algorithm struggles to find the globally optimal solution, leading to premature convergence. To mitigate this issue, this paper introduces an information-sharing mechanism during the staying behavior phase. This mechanism employs a dynamic opposition-based learning strategy and a neighborhood search method to generate populations of different categories based on the original population, thereby broadening the variety and scope of the search population. This enhancement helps the algorithm escape local optima and improves its overall performance.

The dynamic opposition-based learning (DOL) mechanism is derived from the foundational principles of opposition-based learning (OBL) [31]. In OBL, the diversity of a population is enhanced by generating opposite points of the current solutions within the search space. Building upon this, the DOL mechanism not only retains the capability to generate opposite solutions but also incorporates a dynamic factor. This factor introduces a higher degree of randomness into the location where the opposite solution is generated, moving beyond the fixed symmetry of standard OBL. The process for generating an opposite solution using the DOL mechanism is implemented via Equation (42). The new population created through this process is designated as the OL population.

(41) $${\vec{X}}_{f}\left(t\right)=ub+lb-\vec{X}\left(t\right)$$

(42) $${\vec{X}}_{reverse}\left(t\right)=\vec{X}\left(t\right)+\varphi \cdot rand\cdot \left(rand\cdot {\vec{X}}_{f}-\vec{X}\left(t\right)\right)$$

where *X_f_* represents the traditional reverse solution, *X*_*reverse*_(*t*) represents the individuals in the dynamic reverse population, and *X*(*t*) represents the individuals in the original population. *t* is the number of iterations. *φ* represents the dynamic weight factor, which is used to control the generation intensity of the reverse solution.

This paper sets the dynamic weight factor *φ* as shown in Equation (43). Through nonlinear dynamic adjustment, the algorithm focuses on global search in the early iterations and on local development in the later iterations.

(43) $$\varphi =0.5\cdot {\left(1+\cos\left(\pi \cdot \frac{t}{MAX_{Iter}}\right)\right)}^{2}$$

To effectively enhance the local exploration ability of the algorithm and the diversity of the population, this paper also introduces a neighborhood search strategy [32]. The generation of the neighborhood search population is achieved by Formula (44), and the newly generated population is named the *RD* population.

(44) $${\vec{X}}_{rd}\left(t\right)=\vec{X}\left(t\right)+\frac{\left|ub-lb\right|}{2\cdot N\cdot rand\cdot sign\left(rand-0.5\right)}$$

where *X*_*rd*_ represents an individual in the neighborhood search group, and *sign* represents the sign function.

After generating two new populations, the original population, the *OL* population and the *RD* population are merged into a temporary population of size *3N*. Using the elite selection strategy, the fitness of all the generated individuals is evaluated, and the top *N* individuals with the best fitness are retained as the new population.

#### 2.3.3. Analysis of Algorithm Complexity

(1) Temporal Complexity Analysis

Compared with the standard PO algorithm, MIPO incorporates Chebyshev chaotic map initialization, which exhibits a temporal complexity of *O(N⋅D)*. The computational overhead for updating adaptive weight parameters *ω*_1_, *ω*_2_, and related parameters for *N* individuals is *O(N)*. The dynamic information sharing mechanism triggered during the “staying behavior” generates temporary populations and executes sorting-based selection, incurring a temporal complexity of *O (N⋅D+N log N)*. Collectively, the overall temporal complexity of MIPO is *O (T⋅ (N⋅D+N log N))*. Although this introduces an additional *O (N log N)* sorting overhead compared to the standard PO, this constitutes a reasonable computational cost for the substantial performance enhancement achieved. Furthermore, its dominant complexity *O(T⋅N⋅D)* remains of the same order as mainstream algorithms such as PSO and GWO, rendering the computational efficiency acceptable.

(2) Spatial Complexity Analysis

In comparison with the standard PO, MIPO requires additional storage for adaptive parameters (e.g., *ω*_1_, *ω*_2_, *φ*), contributing a constant spatial complexity of *O(1)*. When the dynamic information sharing mechanism is activated, temporary storage for the *OL* population and *RD* population is required, resulting in a peak spatial complexity of *O(3N⋅D)*. Overall, the fundamental spatial complexity of MIPO remains *O(N⋅D)*, with memory consumption predominantly governed by the linear relationship with population size *N* and problem dimensionality *D*. The memory requirements are explicit and controllable, enabling adaptation to large-scale optimization problems with favorable scalability.

#### 2.3.4. Algorithm Pseudocode

Based on the previous content, the pseudo-code of the MIPO algorithm has been organized as follows in Algorithm 1, which illustrates the operation logic of MIPO as well as the collaborative relationship between the mathematical model and each process.
**Algorithm 1: MIPO**1.**Initialize** the MIPO parameters2.**Initialize** parrot *i* positions *X_i_* based on Chebyshev chaotic mapping3.**For** *i =* 1: MAX_Iter_ **do**4.     Calculate the fitness function5.     Find the best position6.     Boundary check and correction7.     **For** *j* = 1:*N*
**do**8.     *St* = *randi*([1,4])9.          Behavior 1: The foraging behavior10.          **If** *St* == 1 **Then**11.               Update position with Formula (31)12.          Behavior 2: The staying behavior13.          **Elseif** *St* == 2 **Then**14.               **for** each individual in population **do**15.               Update the dynamic weighting factor with Formula (43)16.               Generate the *OL* population and the *RD* population using Formulas (42) and (44).17.               Calculate the fitness of each population and store these populations in a new vector.18.               **end for**
19.               Sort all three populations according to the fitness20.               Select the MAX_Iter_ best individuals as population_new21.          Behavior 3: The communicating behavior22.          **Elseif** *St* == 3 **Then**23.               Update the weighting factor with Formula (39)24.               Update position with Formula (40)25.          Behavior 4: The fear of strangers’ behavior26.          **Elseif** *St* == 4 **Then**27.               Update position with Formula (36)28.          **End if**
29.          **End for**
30.     **End for**
31.Return the best solution

### 2.4. MIPO-ARKEKF Overall Process and Pseudocode

**Figure 5:**
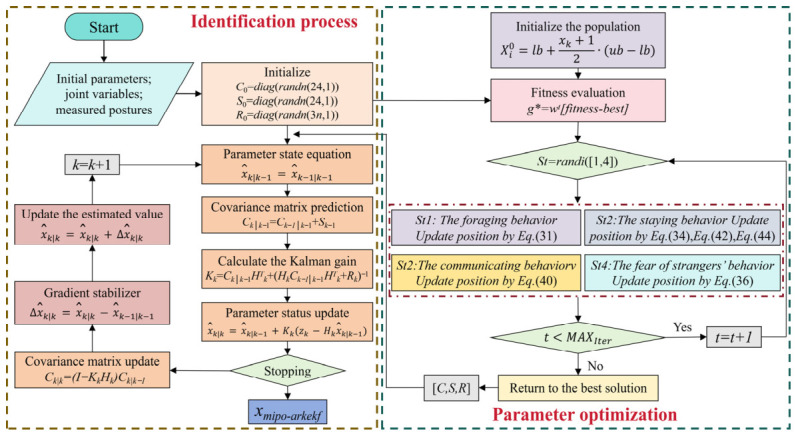
Identification process based on MIPO-ARKEKF.

Based on the previous content, the pseudo-code of MIPO-ARKEKF was compiled as shown in Algorithm 2, which demonstrates the operation logic of MIPO-ARKEKF. On this basis, the algorithm flow visualization as shown in Figure 5 was further established. This visualization provides an intuitive understanding of MIPO-ARKEKF and highlights the collaborative interaction among multiple processes in the algorithm’s mathematical model.
**Algorithm 2:** MIPO-ARKEKF **Input:** *x*_0_, {*Q*_n_}, {$P_{L}^{S}(n)$}**Operation**1.**Initialize** *K*: The number of iterations2.**Initialize** *N*: The number of sample points3.**Initialize** *C*_0_ = diag(randn(24,1)), *S*_0_ = diag(randn(24,1)), *R*_0_ = diag(randn(3n,1))**/--Note: MIPO-Step-/**4.     [*C*, *S*, *R*] = MIPO (*x*_0_, {Q_n_}, {$P_{L}^{S}(n)$}, *C*_0_, *S*_0_, *R*_0_)5.     **for** k = 1 to *K*6.          **for** i = 1 to *N*7.          Update the state according to Formula (16). 8.          Carry out covariance prediction according to Formula (18). 9.          Make *H_k_* evolve with the J part of Formula (10).10.          Calculate the observed value of the position error according to Formula (19). 11.          Calculate the Kalman gain according to Formula (20). 12.          Update the parameter status according to Formula (21). 13.          Update the covariance according to Formula (22). 14.          *x*_mipo-arkekf_ =$\;{\hat{x}}_{k|k}$
15.          Perform the incremental compensation for state estimation according to Formula (24). 16.          Calculate the new state estimation increment based on Formula (25). 17.          **end for**
18.     *k* = *k* + 119.     **end for**
20.**Output:** *x*_mipo-arkekf_

## 3. Results

### 3.1. Performance Testing of the MIPO Algorithm

#### 3.1.1. Experimental Setup and Test Function

This section presents a comparative evaluation of the MIPO algorithm against five other classical algorithms—the whale optimization algorithm (WOA) [33], Harris hawks optimization (HHO) [34], Greylag goose optimization (GGO) [35], the snow geese algorithm (SGA) [36], and hippopotamus optimization (HO) [37]—using the CEC 2022 benchmark suite. The population size is set to 100, and the maximum number of iterations is 500. Since all algorithms employ self-adaptive parameter strategies, no detailed parameter tuning is performed. The simulation experiments are conducted in the MATLAB R2024b environment, running on a system with an Intel(R) Core (TM) i7-14650HX processor, Windows 11 operating system, 32 GB of physical memory, and a processor base frequency of 2.2 GHz. The key characteristics of the test suite functions are summarized in Table 2.

#### 3.1.2. Benchmark Results on the CEC 2022 Test Suite

The dimension of the test set was set to 10-dimensional (10D), and the experimental results are shown in Table 3. According to Table 3, in the 10D tests of the 12 functions, MIPO achieved the following rankings:

Average Value (Ave.): first place in 10 functions (F1, F2, F3, F4, F7, F8, F9, F10, F11, F12); second place in 2 functions (F5, F6).

Standard Deviation (Std.): first place in six functions (F1, F2, F7, F8, F10, F11); second place in four functions (F3, F5, F6, F12); third place in one function (F4); fifth place in one function (F9);

Execution Time (Time): first place in two functions (F11, F12); second place in two functions (F7, F9); fourth place in six functions (F1, F3, F4, F5, F8, F10); fifth place in one function (F6); sixth place in one function (F2).

Best Value (Best): first place in seven functions (F3, F4, F6, F7, F9, F10, F12); second place in four functions (F1, F2, F8, F11); third place in one function (F5).

**Figure 6:**
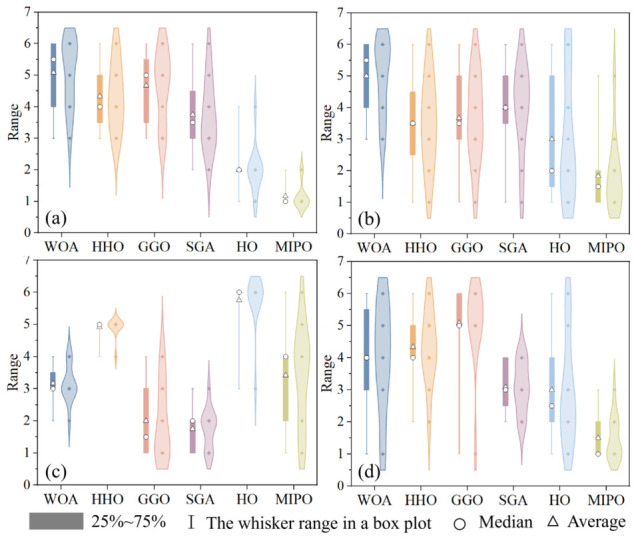
Test result (CEC 2022 10D) ranking: (**a**) Ave.; (**b**) Std.; (**c**) Time; (**d**) Best.

Based on the data in Table 3, Figure 6 visually presents the performance of six algorithms, including four indicators: average value, standard deviation, running time, and best value.

Similarly, the dimension of the test suite was set to 20-dimensional (20D). The experimental results are shown in Table 4. As can be seen from Table 4, in the 20D tests of the 12 functions, the ranking of MIPO is as follows:

Average Value (Ave.): first place in 12 functions (F1, F2, F3, F4, F5, F6, F7, F8, F9, F10, F11, F12);

Standard Deviation (Std.): first place in 10 functions (F1, F2, F4, F6, F7, F8, F9, F10, F11, F12); second place in 1 function (F5); third place in one functions (F3);

Execution Time (Time): first place in four functions (F2, F3, F7, F8); second place in three functions (F1, F5, F6); third place in three functions (F4, F11, F12); fourth place in two functions (F9, F10).

Best Value (Best): first place in 12 functions (F1, F2, F3, F4, F5, F6, F7, F8, F9, F10, F11, F12).

**Figure 7:**
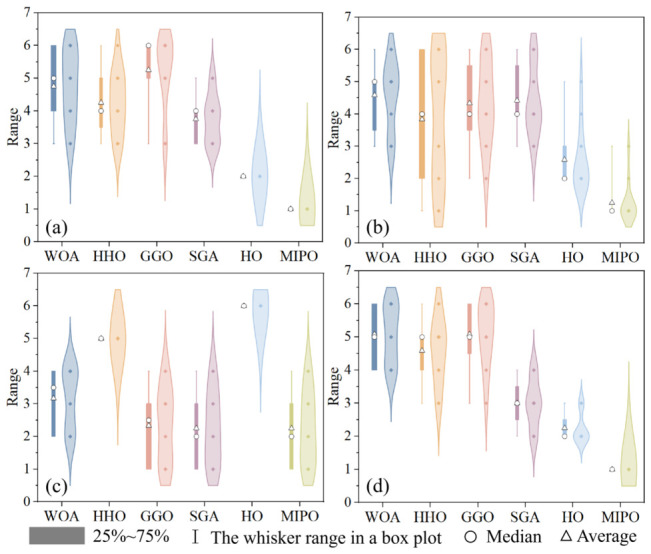
Test result (CEC 2022 20D) ranking: (**a**) Ave.; (**b**) Std.; (**c**) Time; (**d**) Best.

Based on the data in Table 4, Figure 7 visually presents the performance of six algorithms, including four indicators: average value, standard deviation, running time, and best value.

By analyzing the performance of six algorithms in the CEC 2022 test suite, it can be observed that MIPO has a more significant performance advantage. From the ranking visualization (Figure 6 and Figure 7), it can be seen that when the dimension of the test suite changes from 10D to 20D, the ranking of MIPO in terms of average value, variance, time consumption, and best result in the 20D test is significantly better than that in the 10D test. This indicates that MIPO outperforms other algorithms in terms of performance when dealing with high-dimensional problems.

### 3.2. Verification of Error Compensation in Robot Polishing System

#### 3.2.1. General Settings

(1) Evaluative criteria

In the research on robot calibration, the following indicators are commonly used to evaluate the performance of the proposed model, which is specifically represented by Formula (45) [38].

(45) $$\left\{\begin{array}{c} MAX=\underset{1\leq i\leq n}{\max}\left\{\sqrt{{\left(P_{i}-P_{i}^{\prime }\right)}^{2}}\right\},\\ MEAN=\frac{1}{n}\sum_{i=1}^{n}\sqrt{{\left(P_{i}-P_{i}^{\prime }\right)}^{2}},\\ RMSE=\sqrt{\frac{1}{n}\sum_{i=1}^{n}{\left(P_{i}-P_{i}^{\prime }\right)}^{2}}. \end{array}\right.$$

(2) Datasets

In this study, 2000 sets of sample data were uniformly collected throughout the entire workspace of the robotic polishing system depicted in Figure 1. To ensure that the selected measurement points adequately and uniformly cover the representative workspace of polishing operations, spatial filling design principles were adopted. From the 2000 sample sets, 400 representative sample points were systematically selected to achieve comprehensive spatial coverage, thereby ensuring the robustness and generalizability of the calibration model. To mitigate the influence of stochastic factors and computational fluctuations, the entire calibration-comparison process was independently repeated 10 times, and the final reported results were determined by the arithmetic mean or mean ± standard deviation of the 10 runs, depending on the reporting format. Each experiment employed a training-validation ratio of 70%–30%, and the sampled compensation points and validation points remained unchanged across repeated runs.

**Figure 8:**
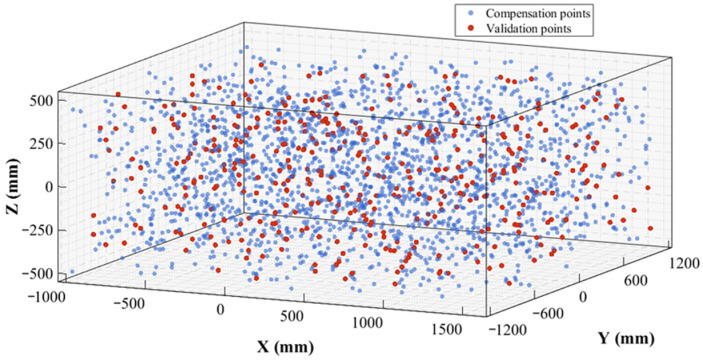
Compensation and validation position in the workspace.

For the calibration comparisons in Section 3.2.2, all methods were evaluated under the same dataset, the same training–validation split, and the same stopping criteria to ensure a fair comparison. For the single identification methods (M1–M5), the maximum number of iterations was fixed at 60 for all models. For the hybrid identification methods, the total iteration budget was also fixed at 60, with 30 iterations assigned to the first-stage algorithm and 30 iterations assigned to the second-stage algorithm. For the MIPO-based optimization, the population size was set to 100, and the search ranges of the adaptive factors were fixed according to Section 2.2.3, i.e., α ∈ [10^−7^, 10^−5^], β ∈ [10^−7^, 10^−5^], and γ ∈ [10^−5^, 10^−3^]. The internal adaptive parameters of MIPO followed the formulations given in Section 2.3.2. Unless otherwise stated, all stochastic optimization runs were performed under identical parameter settings, with only the random seed varying across repeated runs. Figure 8 illustrates the spatial distribution of compensation points and validation points within the robotic motion space. Table 5 presents exemplars of five sample data sets, each comprising the joint angle values of the robot and the corresponding laser tracker measurements.

#### 3.2.2. Model Performance Evaluation

(1) Ablation Study of MIPO-ARKEKF

To evaluate the individual contributions of the gradient stabilizer and the MIPO-based adaptive covariance optimization in the proposed MIPO-ARKEKF framework, an ablation study was conducted. Four configurations were tested under the same calibration dataset and experimental conditions: the baseline EKF, EKF with the gradient stabilizer (EKF+GSBISE), EKF with MIPO-based adaptive covariance optimization (EKF+MIPO), and the complete MIPO-ARKEKF method. The comparison results are summarized in Table 6.

As shown in Table 6, both the gradient stabilizer and the MIPO-based adaptive covariance optimization contribute positively to the performance improvement of the EKF-based identification framework. Compared with the baseline EKF, EKF+GSBISE reduces the MEAN, RMSE, and MAX errors from 0.5514 mm, 0.6194 mm, and 1.4521 mm to 0.5126 mm, 0.5687 mm, and 1.3685 mm, corresponding to reductions of 7.04%, 8.19%, and 5.76%, respectively. This indicates that the introduced gradient stabilizer can effectively improve the stability of the filtering process and alleviate estimation degradation caused by repeated linearization.

By comparison, EKF+MIPO achieves larger improvements than EKF+GSBISE, reducing the MEAN, RMSE, and MAX errors to 0.4689 mm, 0.5213 mm, and 1.3246 mm, respectively, which correspond to reductions of 14.96%, 15.84%, and 8.78% relative to the baseline EKF. These results show that adaptive covariance optimization plays a more significant role in improving the identification accuracy, mainly because it enhances the consistency between the filtering model and the actual measurement uncertainty.

Among all the compared methods, the complete MIPO-ARKEKF framework achieves the best performance, with the MEAN, RMSE, and MAX errors further reduced to 0.4382 mm, 0.4858 mm, and 1.2869 mm, respectively. Relative to the baseline EKF, the overall reductions reach 20.53%, 21.57%, and 11.38%. This result confirms that the two modules are complementary: the gradient stabilizer mainly improves estimation stability, while the MIPO-based adaptive covariance optimization mainly improves noise-matrix matching and filtering accuracy. Their combination yields the best overall identification performance.

It is worth noting that the gain of EKF + GSBISE is lower than that of EKF + MIPO, which is consistent with their different positions in the EKF signal flow. The gradient stabilizer acts on the state update path, compensating for gradient degradation caused by accumulated truncation errors in Taylor linearization. Its essential function is numerical stabilization-preventing iteration stagnation or divergence. However, in static parameter estimation problems such as robot kinematic calibration, where the state equation degenerates, the process and measurement noise covariance matrices become the dominant hyperparameters governing estimation accuracy. EKF+MIPO directly manipulates these matrices, altering the Kalman gain computation and thereby precisely regulating the trust allocation between prediction and observation. This direct parameterization of the filter’s core weighting mechanism yields a stronger marginal gain than the stabilizer’s indirect gradient correction.

(2) Compared with a single identification method

To verify the advantages of the MIPO-ARKEKF algorithm in error compensation for the robot polishing system, this paper conducts a performance comparison analysis of this algorithm with four identification methods. The specific descriptions of the four algorithms are as follows:

M1: The EKF algorithm is commonly used in robot systems to achieve error compensation. It achieves state estimation for nonlinear problems through the first-order Taylor expansion and has been widely applied in practical engineering [9].

M2: The IPSO algorithm is an improvement of the particle swarm optimization algorithm, which solves the problems of parameter adaptive adjustment and the easy local optimum in the PSO algorithm. PSO has been widely applied in engineering problems. Using IPSO can help fully compare the performance differences between MIPO-ARKEKF and meta-heuristic algorithms [14].

M3: LASSO is an improvement based on the least squares (LS) algorithm. It achieves more precise identification performance through a penalty function. LS plays a significant role in error identification and compensation [13].

M4: The UKF algorithm is commonly used for state estimation in nonlinear systems. Its advantage lies in that when nonlinear functions are expanded, higher-order term information can be retained, and it is easy to implement in high-dimensional spaces [10].

M5: The MIPO-ARKEKF algorithm proposed in this paper.

**Figure 9:**
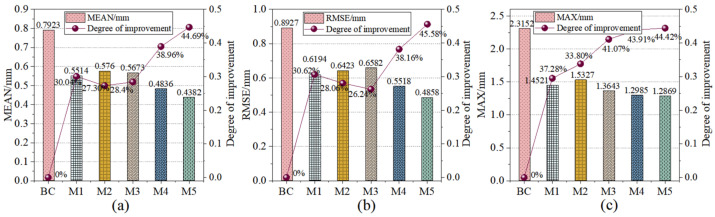
Comparison of compensation performance for M1–M5. (**a**) MEAN error; (**b**) RMSE error; (**c**) MAX error.

After 60 iterations of the aforementioned five models (M1–M5), the identification results were aggregated for a comparative analysis to evaluate the performance of each algorithm in robotic error compensation. Table 7 summarizes the compensation outcomes for all models, where “BC” denotes the state before compensation. Based on Table 7, a visualization of the compensation performance comparison for M1 through M5 was implemented, as illustrated in Figure 9. This visualization intuitively demonstrates the performance status of each algorithm and the extent of improvement relative to the pre-compensation state.

**Figure 10:**
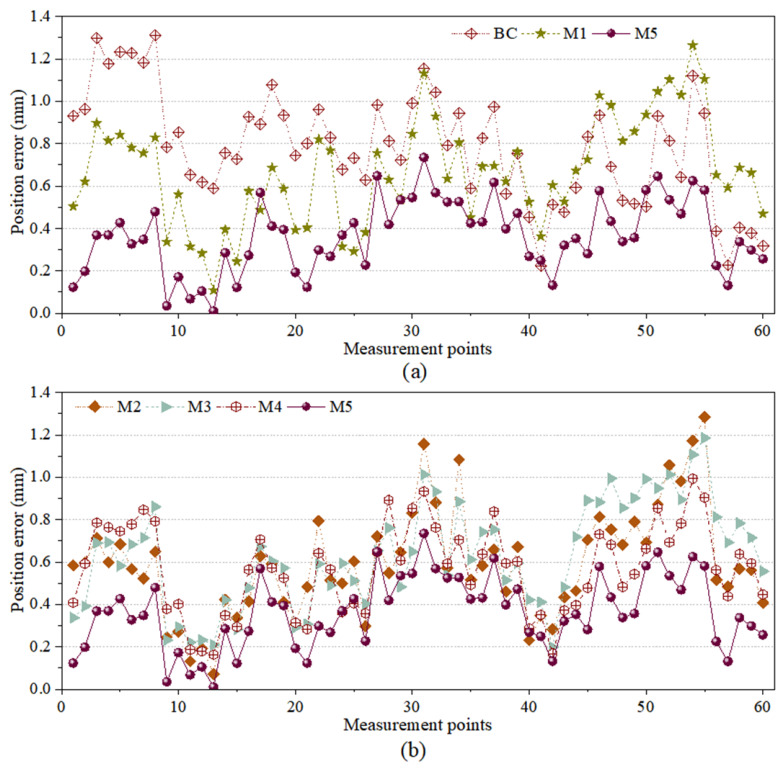
The system positioning errors of the measurement points. (**a**) Before compensation, M1 and M5; (**b**) M2, M3, M4 and M5.

**Figure 11:**
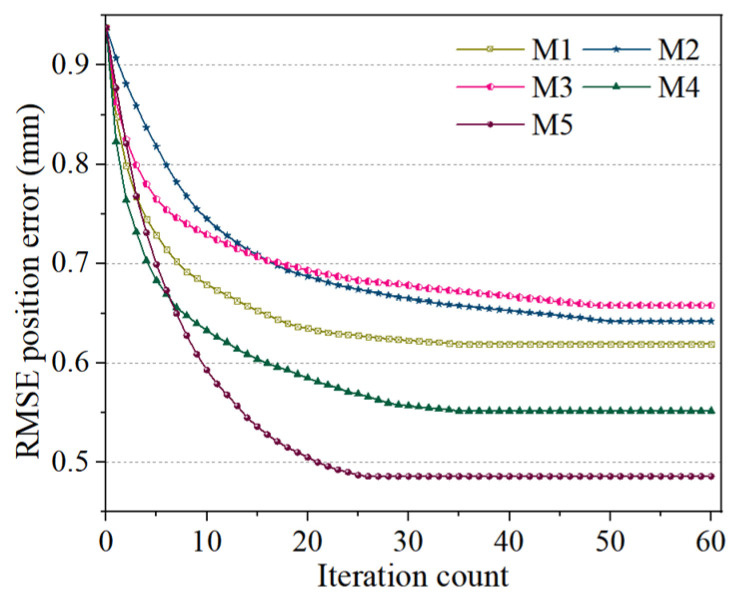
Comparison of convergence curves for M1–M5.

To further investigate the compensation effects of the different models, the absolute positioning accuracy changes before and after compensation were analyzed using the systematic positioning errors of measured points, as depicted in Figure 10. Figure 10a displays the compensation effects of the pre-compensation state, M1, and M5, while Figure 10b presents the effects of M2, M3, M4, and M5. The results validate the accuracy and reliability of the proposed MIPO-ARKEKF algorithm. Furthermore, a comparison of the convergence performance among the models was conducted to analyze their convergence capabilities and variation trends during the iterative process, as shown in Figure 11.

As shown in Table 4 and Figure 9, the errors of the M5 algorithm in MEAN, RMSE and MAX are 0.4382 mm, 0.4858 mm and 1.2869 mm, respectively. Compared with the situation before compensation, these errors have decreased by 44.69%, 45.58% and 44.42%, respectively. Particularly, the M1 algorithm is the standard model of the M5 algorithm. Its errors in MEAN, RMSE and MAX are 0.5514 mm, 0.6194 mm and 1.4521 mm, respectively. The M5 algorithm and the M1 algorithm have decreased the errors in each aspect by 20.53%, 21.57% and 11.38%, respectively. This proves that the adaptive strategy using MIPO and the gradient stabilizer proposed in this paper for improving the performance of the standard EKF algorithm are effective.

When compared with other algorithms, the RMSE error of the M5 model decreased by 24.43%, 26.19%, and 11.96%, respectively, compared to the M2, M3, and M4 algorithms. The MEAN error decreased by 23.92%, 22.76%, and 9.39%, respectively. The MAX error also showed a similar trend. This proves that the M5 algorithm has higher identification accuracy compared to other compensation algorithms.

In terms of algorithm convergence, the M5 algorithm exhibits the best convergence speed and performance. It can achieve the optimal error compensation with a relatively small number of iterations. During the first 25 iterations, the RMSE error of the M5 algorithm rapidly decreases and stabilizes at 0.4858 mm. Although in the first 10 iterations, the convergence speed of other models is faster, it takes a higher number of iterations to reach stability. However, after reaching stability, the M5 algorithm still maintains the lowest error level, demonstrating its efficiency and stability.

(3) Compared with the hybrid identification method

In the research of error compensation, apart from using a single compensation algorithm, some researchers also employed a hybrid identification method by combining multiple algorithms to achieve higher compensation accuracy. Therefore, this paper will compare the proposed MIPO-ARKEKF algorithm with the hybrid algorithms, further verifying the performance of the MIPO-ARKEKF algorithm. This paper selects two hybrid algorithms for comparative analysis with MIPO-ARKEKF. The specific descriptions of the two algorithms are as follows.

LM+GA: This method first uses the LM algorithm for initial recognition to obtain the initial estimated values of the parameters. Then, the GA is introduced for global optimization to correct the local optimal problem that may be caused by the LM algorithm’s sensitivity to the initial values, thereby achieving a comprehensive and precise identification of all kinematic parameters [12].

EKF+DQPSO: This method first employs EKF for the initial parameter estimation. To enhance the performance of the EKF algorithm, a specially designed DQPSO strategy is developed to optimize the kinematic parameter errors estimated by the EKF initially, in order to achieve higher accuracy in parameter identification [15].

MIPO-ARKEKF: The algorithm proposed in this paper optimizes the standard EKF algorithm based on the adaptive strategy of MIPO and the gradient stabilizer, in order to enhance the error identification and compensation performance.

**Figure 12:**
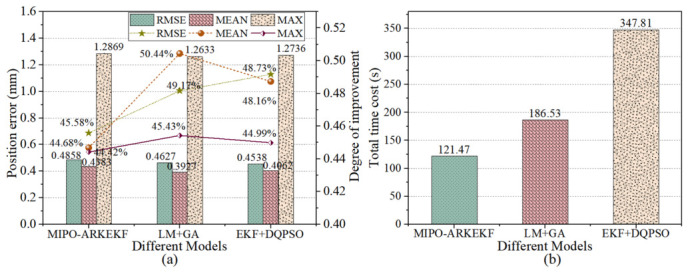
Compensation performance of the three methods. (**a**) Position error; (**b**) Time cost.

Similarly, each of the three methods was subjected to 60 iterations, and the iterative results were aggregated for subsequent comparative analysis. Unlike the single method, in the hybrid method, the preceding and subsequent algorithms were executed for 30 iterations each. Figure 11 illustrates the error compensation accuracy and the computational time cost of the three methods, while Figure 12 presents their convergence curves. By comparing with the hybrid methods, a more in-depth assessment of the precision and efficiency of the MIPO-ARKEKF algorithm in error identification and compensation can be achieved.

As illustrated in Figure 12a, the MIPO-ARKEKF algorithm achieves compensation performance comparable to that of LM+GA and EKF+DQPSO. The RMSE (root mean square error) of the MIPO-ARKEKF algorithm is 0.4858 mm, while those of EKF+DQPSO and LM+GA are 0.4538 mm and 0.4627 mm, respectively. Compared with the other two methodologies, the MIPO-ARKEKF algorithm exhibits differences of merely 0.032 mm and 0.0231 mm. This demonstrates that the MIPO-ARKEKF algorithm maintains a leading position in terms of compensation accuracy.

**Figure 13:**
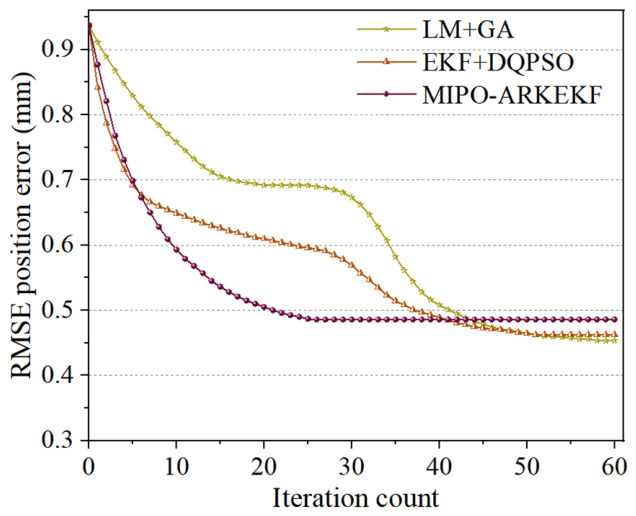
Comparison of convergence of three methods.

Regarding computational time consumption, the MIPO-ARKEKF algorithm demonstrates substantial advantages. As depicted in Figure 12b, the total computational time for the MIPO-ARKEKF algorithm is 121.47 s. The LM+GA method consumes 186.53 s, whereas EKF+DQPSO requires 347.81 s. The MIPO-ARKEKF algorithm reduces time consumption by 34.88% and 65.08%, respectively. Furthermore, as shown in Figure 13, the MIPO-ARKEKF algorithm exhibits superior convergence rate characteristics, achieving convergence within 25 iterations, while EKF+DQPSO and LM+GA require more than 50 iterations to reach convergence.

In summary, the MIPO-ARKEKF algorithm achieves compensation accuracy comparable to hybrid methodologies while exhibiting distinct advantages in computational time consumption and convergence rate. These characteristics establish a solid foundation for its future application in scenarios requiring periodic online calibration or adaptive compensation. This adequately demonstrates the reliability and developmental potential of the MIPO-ARKEKF algorithm.

To further verify whether the observed RMSE advantages of M5 over both the single identification methods and the hybrid identification methods are statistically meaningful rather than caused by random variation, a statistical significance analysis was performed based on the repeated experimental results reported above. Specifically, all compared methods were independently executed 10 times under the same calibration dataset and stopping criteria, and the RMSE values obtained from these repeated runs were collected for pairwise comparison. M5 was then compared with BC, M1–M4, LM+GA, and EKF+DQPSO, respectively, using an independent-samples t-test. The mean RMSE differences together with the corresponding *p*-values are summarized in Table 8.

As shown in Table 8, the RMSE reductions achieved by M5 are statistically significant in all comparison pairs (*p* < 0.05). These results further confirm that the performance gain of M5 is stable across repeated runs rather than caused by occasional favorable results.

The statistically validated reduction in calibration error provides the basis for the subsequent improvement in polishing-path tracking and practical polishing quality, which will be further analyzed in Section 3.2.3 and Section 3.2.4.

#### 3.2.3. Verification of Polishing Path Compensation

**Figure 14:**
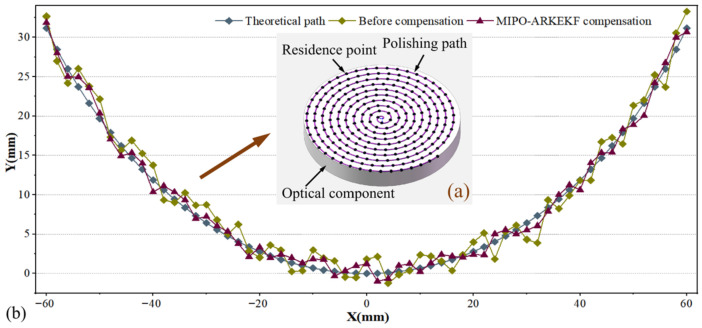
Comparison of polishing path before and after compensation. (**a**) Polished trace of residence; (**b**) Trajectory tracking errors of different algorithms.

To validate the effectiveness of the proposed MIPO-ARKEKF algorithm, it was implemented in a robotic polishing system. Figure 14a depicts the polishing trajectory executed by the robot. Figure 14b records the theoretical path of the optical component polishing, the actual path before compensation, and the path after compensation using the MIPO-ARKEKF algorithm. The results demonstrate that the compensated path aligns significantly more closely with the theoretical trajectory, confirming the practical efficacy of the algorithm in enhancing positioning accuracy. Although the trajectory points on the optical lens are also positions in the robot workspace, they represent a task-specific subset associated with the actual polishing path rather than a globally distributed validation set.

**Figure 15:**
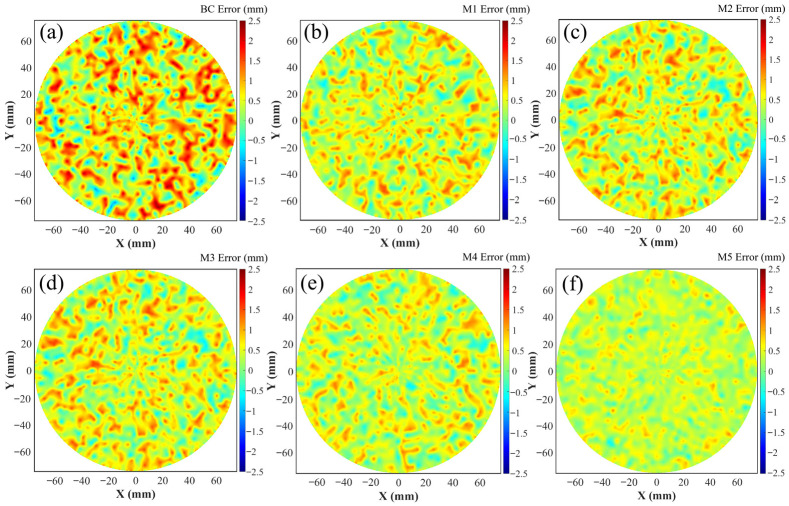
Comparison of error distribution at the residence points. (**a**) Before compensation; (**b**) M1 compensation; (**c**) M2 compensation; (**d**) M3 compensation; (**e**) M4 compensation; (**f**) M5 compensation.

Figure 15 provides a detailed comparison of the compensation effects achieved by different algorithms at the dwell points. In Figure 15a, which illustrates the deviation before compensation, the area is characterized by a darker color and a larger size. Following compensation, a significant reduction in the dwell point deviations is observed, evidenced by a noticeable lightening of the color. However, the degree of improvement, reflected in the final color depth and area size, varies among the algorithms. Figure 15b, Figure 15c, Figure 15d, and Figure 15e display the results after compensation by the M1, M2, M3, and M4 algorithms, respectively. While all show substantial improvement compared to the uncompensated state, considerable error ranges remain. In contrast, as shown in Figure 15f, the area after compensation using the MIPO-ARKEKF algorithm exhibits the lightest color, indicating that the deviations at the dwell points have been minimized to the greatest extent. This result demonstrates the superior effectiveness of the MIPO-ARKEKF algorithm in compensating for geometric errors within the robotic polishing system, thereby significantly enhancing the tracking accuracy of the polishing path.

As summarized in Table 9, all compensation methods improve the tracking accuracy of the polishing path compared with the uncompensated case (BC), confirming that geometric calibration effectively enhances the execution accuracy of task-specific trajectory points. Among all the compared methods, M5 achieves the best overall performance.

Specifically, the proposed M5 method reduces the MEAN, RMSE, and MAX tracking errors from 0.847 mm, 0.963 mm, and 2.184 mm in the uncompensated case to 0.438 mm, 0.498 mm, and 1.241 mm, corresponding to reductions of 48.29%, 48.29%, and 43.18%, respectively. In addition, the proportion of trajectory points within the ±0.5 mm error band increases from 12.5% to 68.5%, while the proportion within the ±1.0 mm error band rises from 45.3% to 96.2%. These results indicate that the proposed method not only reduces the average trajectory deviation, but also significantly improves the practical accuracy pass rate of the polishing dwell points.

Compared with representative UKF-based method M4, M5 further reduces the MEAN, RMSE, and MAX errors by 14.45%, 15.02%, and 2.59%, respectively. Meanwhile, the proportion of points within the ±0.5 mm and ±1.0 mm error bands increases by 16.2 and 4.5 percentage points, respectively. This shows that the advantage of the proposed calibration framework is preserved when transferred from global workspace compensation to the task-specific polishing path.

Overall, the results in Table 9 provide a direct kinematic explanation for the subsequent polishing-quality improvement. More accurate tracking of the polishing path means that the actual dwell-point execution is closer to the theoretical removal path, which helps the material removal distribution better match the planned polishing process and ultimately contributes to the lower PV and RMS values observed in the practical polishing experiments.

#### 3.2.4. Polishing Experiment Verification

**Figure 16:**
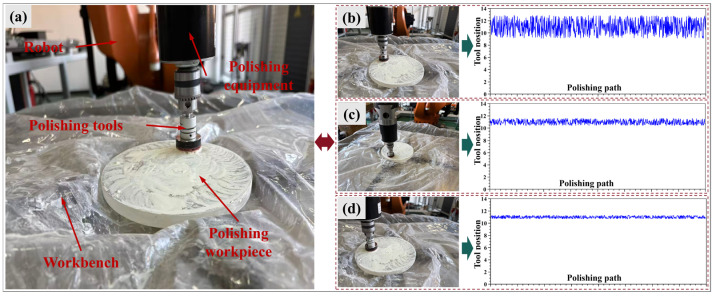
Schematic diagram of the polishing experiment. (**a**) Experimental setup; (**b**) before compensation; (**c**) M4 compensation; (**d**) M5 compensation.

In order to verify the effectiveness of the MIPO-ARKEKF algorithm in actual polishing processes, experiments were conducted in an actual robotic polishing system. Figure 16a illustrates the robotic polishing experimental platform. In this phase, the actual positions of the robot end-effector along the dwell points of the optical component were acquired. The discrepancy between the theoretical path and the actual path was compared to validate whether the integration of the compensation algorithm enhances path tracking accuracy. Subsequently, the polishing experiments were compensated using the representative UKF algorithm (denoted as M4) and the proposed MIPO-ARKEKF algorithm (denoted as M5), respectively. Positional data under different compensation strategies were collected to verify the effectiveness and reliability of the proposed method. The experiments were conducted on an ultra-low-expansion (ULE) glass lens through three polishing trials. To mitigate the influence of extraneous factors, all experiments are performed under consistent processing conditions. The specific parameters of the polishing experiment are shown in Table 10.

Figure 16b, Figure 16c, and Figure 16d present the acquired positional data for selected tool locations under the uncompensated state, M4 compensation, and M5 compensation, respectively. The data reveal a substantial positioning error in the uncompensated case. After applying the M4 algorithm, the error is effectively reduced. With the M5 algorithm, the positioning accuracy of the tool end is significantly improved. These results clearly demonstrate that algorithmic compensation enhances the robot’s absolute positioning accuracy, thereby indirectly improving the tracking precision of the polishing trajectory.

**Figure 17:**
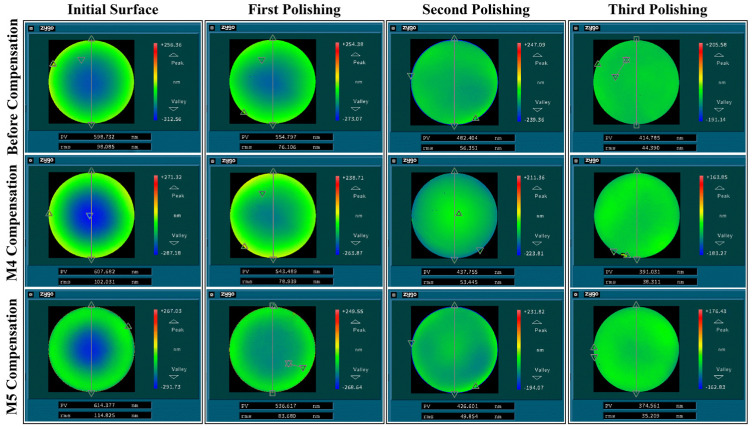
Summary of polishing experiment results.

**Figure 18:**
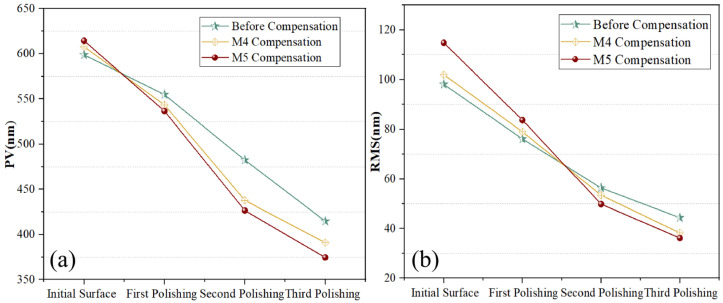
Comparison of PV and RMS at different polishing stages. (**a**) PV, (**b**) RMS.

As summarized in Figure 17, the polishing results obtained using different compensation strategies are presented, with measurements acquired through a 3D profilometer. Furthermore, the PV (peak-to-valley) and RMS (root mean square) values for the three polishing iterations are compiled in Figure 18. The results demonstrate that after the third polishing iteration, the PV value of the ULE lens compensated by the M5 algorithm reaches 374.561 nm, representing a 9.70% reduction compared with the uncompensated value of 414.785 nm. Similarly, the RMS value achieved by M5 compensation is 35.209 nm, which is 20.68% lower than the uncompensated value of 44.390 nm. For the competing M4 algorithm, the compensated PV value is 391.031 nm and the RMS value is 38.311 nm. Although the M4 algorithm also achieves favorable results, these values are 4.21% and 8.09% higher than those obtained by M5, respectively.

This polishing-quality improvement is consistent with the calibration results and the path-compensation results reported in Section 3.2.2 and Section 3.2.3. In robotic polishing, the final material removal distribution depends directly on the accuracy of dwell-point tracking and tool-path execution. Therefore, the reduction in geometric calibration error improves the consistency between the planned polishing trajectory and the actual motion of the tool center point. More specifically, the lower path-tracking errors achieved by the MIPO-ARKEKF algorithm suppress local over-polishing and under-polishing caused by isolated positioning deviations, thereby enabling the actual material removal process to better match the theoretical removal map. Accordingly, the MIPO-ARKEKF algorithm improves not only the calibration metrics and path-tracking statistics, but also the final PV and RMS performance in the practical polishing experiments.

## 4. Conclusions

This study proposed a calibration method for robotic polishing systems based on a multi-strategy improved parrot optimization algorithm and an adaptive residual extended Kalman filter, termed MIPO-ARKEKF. A kinematic model and a geometric error model of the six-axis robot were first established using the MD-H method, providing the basis for geometric parameter identification and compensation. On this basis, two improvements were introduced into the standard EKF framework: a gradient stabilizer based on state-estimation increments to mitigate degradation caused by repeated linearization, and an adaptive covariance optimization strategy in which the proposed MIPO algorithm was used to adjust the process and measurement noise matrices under practical measurement uncertainty.

The proposed MIPO algorithm incorporated Chebyshev chaotic initialization, adaptive weight adjustment, and a dynamic information sharing mechanism. The benchmark results on the CEC 2022 test suite showed that MIPO achieved competitive optimization performance in both 10D and 20D problems, with particularly stable overall behavior in the higher-dimensional setting. These results indicate that the algorithm is suitable for the covariance-optimization problem involved in robot calibration, which is continuous, nonlinear, and coupled.

The effectiveness of the complete MIPO-ARKEKF framework was further verified through robot calibration experiments. The ablation study showed that both the gradient stabilizer and the MIPO-based adaptive covariance optimization contribute positively to the final performance, and that their combination produces the best overall results. Compared with the baseline EKF, the complete framework reduced the MEAN, RMSE, and MAX positioning errors from 0.5514 mm, 0.6194 mm, and 1.4521 mm to 0.4382 mm, 0.4858 mm, and 1.2869 mm, respectively, corresponding to reductions of 20.53%, 21.57%, and 11.38%. In addition, the repeated-run statistical analysis indicated that the RMSE improvements over the compared methods were statistically significant, supporting the robustness of the observed performance gain.

In comparisons with representative single identification methods, including EKF, IPSO, LASSO, and UKF, the proposed method achieved the lowest overall calibration errors among the tested models. In comparisons with representative hybrid methods, including LM+GA and EKF+DQPSO, the proposed method maintained comparable compensation accuracy while requiring less computational time. Specifically, the computation time was reduced by 34.88% to 65.08%, and convergence was reached within 25 iterations, whereas the compared hybrid methods required more than 50 iterations to approach stability. These results suggest that the proposed method provides a reasonable balance between calibration accuracy and computational efficiency for practical robotic polishing applications.

The practical relevance of the proposed method was also examined through polishing-path compensation and actual polishing experiments. After compensation, the executed polishing trajectory was closer to the theoretical path, and the tracking accuracy at the dwell points was improved. For the polishing path, the MEAN, RMSE, and MAX tracking errors were reduced from 0.847 mm, 0.963 mm, and 2.184 mm to 0.438 mm, 0.498 mm, and 1.241 mm, respectively. At the same time, the proportions of trajectory points within the ±0.5 mm and ±1.0 mm error bands increased substantially. The polishing experiments on ULE glass further showed that the improvement in calibration and path tracking could be transferred to the practical polishing process.

Overall, the results indicate that the proposed MIPO-ARKEKF method is effective for improving geometric error calibration accuracy in robotic polishing systems and can provide competitive compensation performance with relatively favorable computational efficiency. The study also suggests that adaptive covariance optimization is useful for improving the consistency between the filtering process and practical measurement conditions, thereby enhancing the robustness of calibration in real experimental settings.

Several limitations should nevertheless be acknowledged. First, the present framework mainly addresses geometric parameter errors under relatively controlled polishing conditions, whereas dynamic effects such as thermal drift, structural compliance, and contact-induced deformation were not explicitly modeled. Second, although the adaptive covariance strategy improves robustness under practical measurement uncertainty, the achievable calibration performance still depends on the quality, stability, and workspace coverage of the external measurement system. Third, the current implementation is primarily oriented toward offline calibration and compensation, and its applicability to fully online identification and correction remains to be further studied.

Future work may proceed in three directions. First, lighter adaptive strategies may be developed to further reduce computational burden and support faster online calibration. Second, the present framework may be integrated with force/position hybrid control or impedance control to address dynamic errors during polishing. Third, the methodology may be extended to broader robotic precision-manufacturing scenarios and evaluated under more complex operating conditions.

## Figures and Tables

**Table 1:** Geometric parameters of KUKA KR 210 R2700 robot.

Joint i	$\alpha_{i}$/(°)	ai/(mm)	di/(mm)	$\theta_{i}$/(°)
1	−90	350	675	0
2	0	1150	0	−90
3	90	41	0	180
4	−90	0	1200	0
5	90	0	0	0
6	0	0	215	0

**Table 2:** Details of the CEC 2022.

Type	No.	Functions	Dimension	$F_{min}$
Unimodal	1	Shifted and full Rotated Zakharov Function	10 & 20	300
Multimodal	2	Shifted and full Rotated Rosenbrock Function	10 & 20	400
	3	Shifted and full Rotated Expanded Schaffer F6 Function	10 & 20	600
	4	Shifted and full Rotated Non-Continuous Rastrigin Function	10 & 20	800
	5	Shifted and full Rotated Levy Function	10 & 20	900
Hybrid	6	Hybrid Function 1 (N = 3)	10 & 20	1800
	7	Hybrid Function 2 (N = 6)	10 & 20	2000
	8	Hybrid Function 3 (N = 5)	10 & 20	2200
Composition	9	Composition Function 1 (N = 5)	10 & 20	2300
	10	Composition Function 2 (N = 4)	10 & 20	2400
	11	Composition Function 3 (N = 5)	10 & 20	2600
	12	Composition Function 4 (N = 6)	10 & 20	2700

**Table 3:** Results of the CEC 2022(10D).

Function	WOA				HHO			
	Ave.	Std.	Time	Best	Ave	Std	Time	Best
F1	4.0633 × 10^4^	1.5639 × 10^4^	4.3139 × 10^−3^	1.9089 × 10^4^	8.5142 × 10^3^	1.7701 × 10^3^	7.8563 × 10^−3^	3.6263 × 10^3^
F2	5.4567 × 10^2^	1.1998 × 10^2^	4.5891 × 10^−3^	4.0896 × 10^2^	6.5367 × 10^2^	2.0036 × 10^2^	7.0144 × 10^−3^	4.4193 × 10^2^
F3	6.4435 × 10^2^	1.4294 × 10^1^	6.3673 × 10^−3^	6.1951 × 10^2^	6.4601 × 10^2^	1.1603 × 10^1^	1.3606 × 10^−2^	6.1656 × 10^2^
F4	8.6055 × 10^2^	1.7902 × 10^1^	4.6168 × 10^−3^	8.3096 × 10^2^	8.2965 × 10^2^	8.2892 × 10^0^	9.0629 × 10^−3^	8.1132 × 10^2^
F5	1.7175 × 10^3^	6.3301 × 10^2^	4.6328 × 10^−3^	9.8960 × 10^2^	1.4931 × 10^3^	2.0629 × 10^2^	9.7785 × 10^−3^	1.0564 × 10^3^
F6	2.7891 × 10^5^	5.1807 × 10^5^	3.8848 × 10^−3^	4.7387 × 10^3^	2.5009 × 10^5^	1.2216 × 10^6^	7.7218 × 10^−3^	2.8399 × 10^3^
F7	2.1145 × 10^3^	4.2134 × 10^1^	7.0025 × 10^−3^	2.0355 × 10^3^	2.0990 × 10^3^	3.2186 × 10^1^	1.5695 × 10^−2^	2.0412 × 10^3^
F8	2.2502 × 10^3^	2.7090 × 10^1^	8.5501 × 10^−3^	2.2178 × 10^3^	2.2408 × 10^3^	1.3560 × 10^1^	1.8885 × 10^−2^	2.2250 × 10^3^
F9	2.6798 × 10^3^	4.6964 × 10^1^	7.7258 × 10^−3^	2.5595 × 10^3^	2.6952 × 10^3^	3.3896 × 10^1^	1.4461 × 10^−2^	2.6131 × 10^3^
F10	2.7038 × 10^3^	3.5295 × 10^2^	5.7623 × 10^−3^	2.5007 × 10^3^	2.6814 × 10^3^	2.3477 × 10^2^	1.3210 × 10^−2^	2.5009 × 10^3^
F11	3.7816 × 10^3^	6.5455 × 10^2^	8.7439 × 10^−3^	2.8174 × 10^3^	3.4464 × 10^3^	4.8285 × 10^2^	1.7899 × 10^−2^	2.7731 × 10^3^
F12	2.9381 × 10^3^	5.9447 × 10^1^	9.3346 × 10^−3^	2.8680 × 10^3^	2.9725 × 10^3^	7.2910 × 10^1^	2.0251 × 10^−2^	2.8728 × 10^3^
**Function**	**GGO**				**SGA**			
	**Ave.**	**Std.**	**Time**	**Best**	**Ave**	**Std**	**Time**	**Best**
F1	8.0496 × 10^3^	2.5634 × 10^3^	2.8125 × 10^−3^	1.3869 × 10^3^	1.0249 × 10^4^	7.3186 × 10^3^	3.4864 × 10^−3^	2.9479 × 10^3^
F2	6.9421 × 10^2^	2.4714 × 10^2^	2.7629 × 10^−3^	4.7932 × 10^2^	4.9619 × 10^2^	6.1795 × 10^1^	3.4211 × 10^−3^	4.1109 × 10^2^
F3	6.4575 × 10^2^	1.2426 × 10^1^	5.7884 × 10^−3^	6.2053 × 10^2^	6.3761 × 10^2^	1.1252 × 10^1^	5.5342 × 10^−3^	6.1266 × 10^2^
F4	8.4737 × 10^2^	1.1630 × 10^1^	3.3517 × 10^−3^	8.1961 × 10^2^	8.4188 × 10^2^	1.5299 × 10^1^	3.6723 × 10^−3^	8.1604 × 10^2^
F5	1.5203 × 10^3^	2.0847 × 10^2^	3.7946 × 10^−3^	1.1572 × 10^3^	1.3244 × 10^3^	2.5676 × 10^2^	4.0691 × 10^−3^	9.2756 × 10^2^
F6	5.3365 × 10^6^	9.4563 × 10^6^	2.6672 × 10^−3^	3.2732 × 10^3^	1.4076 × 10^5^	6.2157 × 10^5^	3.2052 × 10^−3^	2.2216 × 10^3^
F7	2.1116 × 10^3^	3.1927 × 10^1^	6.2351 × 10^−3^	2.0591 × 10^3^	2.0819 × 10^3^	3.5673 × 10^1^	6.0831 × 10^−3^	2.0234 × 10^3^
F8	2.2549 × 10^3^	3.9285 × 10^1^	7.6530 × 10^−3^	2.2264 × 10^3^	2.2745 × 10^3^	5.6301 × 10^1^	7.3077 × 10^−3^	2.2223 × 10^3^
F9	2.7045 × 10^3^	4.1056 × 10^1^	6.4535 × 10^−3^	2.5799 × 10^3^	2.6702 × 10^3^	4.3928 × 10^1^	6.5776 × 10^−3^	2.5340 × 10^3^
F10	2.5938 × 10^3^	8.3097 × 10^1^	6.1651 × 10^−3^	2.5028 × 10^3^	2.6117 × 10^3^	2.2285 × 10^2^	5.7390 × 10^−3^	2.5007 × 10^3^
F11	3.4934 × 10^3^	4.5610 × 10^2^	9.4725 × 10^−3^	2.8528 × 10^3^	3.5162 × 10^3^	5.3270 × 10^2^	8.0275 × 10^−3^	2.7884 × 10^3^
F12	2.8935 × 10^3^	2.4163 × 10^1^	9.5410 × 10^−3^	2.8682 × 10^3^	2.8988 × 10^3^	3.8075 × 10^1^	8.2475 × 10^−3^	2.8654 × 10^3^
**Function**	**HO**				**MIPO**			
	**Ave.**	**Std.**	**Time**	**Best**	**Ave**	**Std**	**Time**	**Best**
F1	7.6127 × 10^3^	1.5743 × 10^4^	8.7989 × 10^−3^	2.0310 × 10^3^	3.9339 × 10^3^	1.2934 × 10^3^	4.5796 × 10^−3^	1.5178 × 10^3^
F2	5.6992 × 10^2^	4.4517 × 10^2^	4.4113 × 10^−3^	5.9981 × 10^2^	4.7902 × 10^2^	6.0416 × 10^1^	8.7139 × 10^−3^	4.1011 × 10^2^
F3	6.2635 × 10^2^	1.1837 × 10^1^	1.3519 × 10^−1^	6.0756 × 10^2^	6.2164 × 10^2^	1.1377 × 10^1^	1.1355 × 10^−2^	6.0752 × 10^2^
F4	8.2700 × 10^2^	8.1397 × 10^0^	1.2246 × 10^−1^	8.1180 × 10^2^	8.2556 × 10^2^	9.0032 × 10^0^	8.5669 × 10^−3^	8.0936 × 10^2^
F5	1.1381 × 10^3^	1.9092 × 10^2^	1.2277 × 10^−1^	9.1444 × 10^2^	1.1525 × 10^3^	1.9647 × 10^2^	9.3599 × 10^−3^	9.3870 × 10^2^
F6	8.0134 × 10^3^	1.0976 × 10^4^	1.1892 × 10^−1^	1.9342 × 10^3^	8.7244 × 10^3^	1.1927 × 10^4^	7.9631 × 10^−3^	1.9295 × 10^3^
F7	2.0627 × 10^3^	1.7502 × 10^1^	1.2900 × 10^−1^	2.0493 × 10^3^	2.0266 × 10^3^	1.4839 × 10^1^	6.1395 × 10^−3^	2.0196 × 10^3^
F8	2.2323 × 10^3^	4.8614 × 10^0^	1.3504 × 10^−1^	2.2216 × 10^3^	2.2182 × 10^3^	2.0307 × 10^0^	1.2692 × 10^−2^	2.2178 × 10^3^
F9	2.6292 × 10^3^	4.7753 × 10^1^	1.2895 × 10^−1^	2.5328 × 10^3^	2.6252 × 10^3^	4.7388 × 10^1^	6.4874 × 10^−3^	2.5290 × 10^3^
F10	2.5591 × 10^3^	6.6647 × 10^1^	1.2740 × 10^−1^	2.5131 × 10^3^	2.5439 × 10^3^	5.9938 × 10^1^	6.4669 × 10^−3^	2.5005 × 10^3^
F11	2.8396 × 10^3^	1.4418 × 10^2^	1.3651 × 10^−1^	2.7152 × 10^3^	2.7965 × 10^3^	9.4712 × 10^1^	7.7842 × 10^−3^	2.7282 × 10^3^
F12	2.8905 × 10^3^	3.3754 × 10^1^	1.3657 × 10^−1^	2.8639 × 10^3^	2.8766 × 10^3^	2.6260 × 10^1^	7.6649 × 10^−3^	2.8421 × 10^3^

**Table 4:** Results of the CEC 2022(20D).

Function	WOA				HHO			
	Ave.	Std.	Time	Best	Ave	Std	Time	Best
F1	7.0325 × 10^4^	2.4418 × 10^4^	3.7487 × 10^−3^	3.2581 × 10^4^	6.2132 × 10^4^	1.9040 × 10^4^	9.3306 × 10^−3^	2.9855 × 10^4^
F2	1.0980 × 10^3^	2.6859 × 10^2^	3.5213 × 10^−3^	6.6943 × 10^2^	1.4962 × 10^3^	3.8429 × 10^2^	7.7153 × 10^−3^	9.6876 × 10^2^
F3	6.7671 × 10^2^	1.3371 × 10^1^	9.0859 × 10^−3^	6.4217 × 10^2^	6.7279 × 10^2^	9.8478 × 10^0^	2.1856 × 10^−2^	6.3942 × 10^2^
F4	9.7943 × 10^2^	2.6208 × 10^1^	5.5305 × 10^−3^	9.2246 × 10^2^	9.2230 × 10^2^	1.5901 × 10^1^	1.3013 × 10^−2^	8.8572 × 10^2^
F5	4.9874 × 10^3^	1.8328 × 10^3^	5.3346 × 10^−3^	2.7001 × 10^3^	3.4740 × 10^3^	4.4592 × 10^2^	1.3730 × 10^−2^	2.4263 × 10^3^
F6	2.2990 × 10^8^	2.9853 × 10^8^	4.0491 × 10^−3^	1.1100 × 10^7^	5.8141 × 10^8^	5.8933 × 10^8^	8.6663 × 10^−3^	1.6117 × 10^7^
F7	2.2799 × 10^3^	9.1898 × 10^1^	1.0532 × 10^−2^	2.1425 × 10^3^	2.2331 × 10^3^	7.8465 × 10^1^	2.6136 × 10^−2^	2.1006 × 10^3^
F8	2.4200 × 10^3^	1.3553 × 10^2^	1.2454 × 10^−2^	2.2384 × 10^3^	2.3919 × 10^3^	1.3883 × 10^2^	2.8931 × 10^−2^	2.2351 × 10^3^
F9	2.7440 × 10^3^	8.9102 × 10^1^	1.1441 × 10^−2^	2.5912 × 10^3^	2.8596 × 10^3^	2.3163 × 10^2^	2.4914 × 10^−2^	2.6037 × 10^3^
F10	5.8995 × 10^3^	1.2710 × 10^3^	8.8653 × 10^−3^	2.5311 × 10^3^	5.4415 × 10^3^	1.2597 × 10^3^	2.1486 × 10^−2^	2.5231 × 10^3^
F11	7.4603 × 10^3^	1.0465 × 10^3^	1.4425 × 10^−2^	5.4616 × 10^3^	7.5540 × 10^3^	6.3489 × 10^2^	3.0093 × 10^−2^	6.0654 × 10^3^
F12	3.2053 × 10^3^	1.7677 × 10^2^	1.5733 × 10^−2^	3.0127 × 10^3^	3.4201 × 10^3^	2.3466 × 10^2^	3.6432 × 10^−2^	3.0955 × 10^3^
**Function**	**GGO**				**SGA**			
	**Ave.**	**Std.**	**Time**	**Best**	**Ave**	**Std**	**Time**	**Best**
F1	7.6344 × 10^4^	4.9754 × 10^4^	2.8857 × 10^−3^	3.2575 × 10^4^	5.4946 × 10^4^	2.1259 × 10^4^	3.3792 × 10^−3^	2.3219 × 10^4^
F2	2.0153 × 10^3^	5.2917 × 10^2^	3.2221 × 10^−3^	9.6536 × 10^2^	1.0506 × 10^3^	2.9835 × 10^2^	3.5468 × 10^−3^	6.2360 × 10^2^
F3	6.7944 × 10^2^	1.0799 × 10^1^	8.9635 × 10^−3^	6.6318 × 10^2^	6.7248 × 10^2^	1.5254 × 10^1^	8.8882 × 10^−3^	6.3314 × 10^2^
F4	9.6174 × 10^2^	2.1439 × 10^1^	5.1455 × 10^−3^	9.0380 × 10^2^	9.4831 × 10^2^	2.6354 × 10^1^	6.4912 × 10^−3^	9.0298 × 10^2^
F5	3.6744 × 10^3^	5.4089 × 10^2^	4.8358 × 10^−3^	2.2202 × 10^3^	3.6381 × 10^3^	8.3901 × 10^2^	5.0304 × 10^−3^	2.3340 × 10^3^
F6	7.1027 × 10^8^	5.7158 × 10^8^	3.3662 × 10^−3^	2.1292 × 10^7^	1.2417 × 10^8^	2.4546 × 10^8^	3.7376 × 10^−3^	9.4184 × 10^5^
F7	2.2219 × 10^3^	7.0775 × 10^1^	1.0446 × 10^−2^	2.1078 × 10^3^	2.2305 × 10^3^	7.7728 × 10^1^	9.9981 × 10^−3^	2.0946 × 10^3^
F8	2.3646 × 10^3^	1.3091 × 10^2^	1.2122 × 10^−2^	2.2424 × 10^3^	2.3965 × 10^3^	1.0303 × 10^2^	1.1437 × 10^−2^	2.2383 × 10^3^
F9	3.0022 × 10^3^	2.1937 × 10^2^	1.1709 × 10^−2^	2.6845 × 10^3^	2.7737 × 10^3^	1.4907 × 10^2^	1.0714 × 10^−2^	2.5735 × 10^3^
F10	5.9117 × 10^3^	1.8882 × 10^3^	8.8122 × 10^−3^	2.5226 × 10^3^	5.4508 × 10^3^	1.4844 × 10^3^	8.5131 × 10^−3^	2.5180 × 10^3^
F11	8.1566 × 10^3^	8.6379 × 10^2^	1.6597 × 10^−2^	6.4771 × 10^3^	7.9631 × 10^3^	1.4600 × 10^3^	1.3701 × 10^−2^	5.3902 × 10^3^
F12	3.2526 × 10^3^	1.5509 × 10^2^	1.7070 × 10^−2^	2.9983 × 10^3^	3.1650 × 10^3^	1.5931 × 10^2^	1.5327 × 10^−2^	2.9709 × 10^3^
**Function**	**HO**				**MIPO**			
	**Ave.**	**Std.**	**Time**	**Best**	**Ave**	**Std**	**Time**	**Best**
F1	3.4898 × 10^4^	9.0101 × 10^3^	1.3352 × 10^−1^	1.7353 × 10^4^	1.6348 × 10^4^	8.3327 × 10^3^	3.0834 × 10^−3^	9.0216 × 10^3^
F2	7.4521 × 10^2^	9.8796 × 10^1^	1.3627 × 10^−1^	5.8211 × 10^2^	6.9979 × 10^2^	8.7905 × 10^1^	3.1746 × 10^−3^	5.3968 × 10^2^
F3	6.5817 × 10^2^	1.2154 × 10^1^	1.5456 × 10^−1^	6.3406 × 10^2^	6.4740 × 10^2^	1.1417 × 10^1^	8.2515 × 10^−3^	6.2315 × 10^2^
F4	9.1350 × 10^2^	1.6408 × 10^1^	1.4736 × 10^−1^	8.8073 × 10^2^	8.8425 × 10^2^	1.5434 × 10^1^	5.7683 × 10^−3^	8.5186 × 10^2^
F5	3.0162 × 10^3^	5.0189 × 10^2^	1.3955 × 10^−1^	1.6608 × 10^3^	2.2219 × 10^3^	4.6387 × 10^2^	4.8585 × 10^−3^	1.6569 × 10^3^
F6	7.8403 × 10^6^	5.1941 × 10^6^	1.3438 × 10^−1^	1.3813 × 10^6^	3.8160 × 10^4^	3.7537 × 10^5^	3.4494 × 10^−3^	1.0820 × 10^4^
F7	2.1610 × 10^3^	4.5688 × 10^1^	1.5487 × 10^−1^	2.0881 × 10^3^	2.1523 × 10^3^	3.6471 × 10^1^	9.5521 × 10^−3^	2.0664 × 10^3^
F8	2.2935 × 10^3^	6.2503 × 10^1^	1.6005 × 10^−1^	2.2316 × 10^3^	2.2494 × 10^3^	2.2901 × 10^1^	1.1244 × 10^−2^	2.2193 × 10^3^
F9	2.6402 × 10^3^	6.0322 × 10^1^	1.5817 × 10^−1^	2.5355 × 10^3^	2.5886 × 10^3^	2.6836 × 10^1^	1.6376 × 10^−2^	2.5281 × 10^3^
F10	4.7537 × 10^3^	1.6849 × 10^3^	1.5069 × 10^−1^	2.5009 × 10^3^	3.6860 × 10^3^	1.0864 × 10^3^	8.8878 × 10^−3^	2.5009 × 10^3^
F11	4.7086 × 10^3^	5.5151 × 10^2^	1.6752 × 10^−1^	3.8007 × 10^3^	4.2941 × 10^3^	3.3090 × 10^2^	1.5685 × 10^−2^	3.1851 × 10^3^
F12	3.1569 × 10^3^	1.1132 × 10^2^	1.7283 × 10^−1^	2.9964 × 10^3^	3.0589 × 10^3^	8.7306 × 10^1^	1.6483 × 10^−2^	2.8299 × 10^3^

**Table 5:** Five detailed sample data.

No.	Angle Values of Joints/°						Measurement Values/mm		
	q_1_	q_2_	q_3_	q_4_	q_5_	q_6_	x	y	z
1	33.99	43.73	3.37	86.05	−5.53	233.66	543.57	−369.32	39.59
2	40.81	41.74	2.25	92.13	−6.45	290.45	572.52	−337.32	42.31
3	38.98	45.92	2.37	89.86	−8.45	253.3	562.97	−327.64	40.38
4	41.33	42.57	2.43	90.89	9.09	272.7	537.15	−316.82	38.73
5	37.13	50.57	2.34	87.05	−8.25	235.74	547.46	−341.74	38.46

**Table 6:** Summary of ablation experiment results.

Metrics	EKF	EKF+GSBISE	EKF+MIPO	MIPO-ARKEKF
MEAN/mm	0.5514 ± 0.0165	0.5126 ± 0.0125	0.4689 ± 0.0146	0.4382 ± 0.0114
RMSE/mm	0.6194 ± 0.0186	0.5687 ± 0.0137	0.5213 ± 0.0158	0.4858 ± 0.0126
MAX/mm	1.4521 ± 0.0436	1.3685 ± 0.0313	1.3246 ± 0.0335	1.2869 ± 0.0283

**Table 7:** Compensation results of all models.

Metrics	BC	M1	M2	M3	M4	M5
MEAN/mm	0.7923 ± 0.0317	0.5514 ± 0.0165	0.5760 ± 0.0202	0.5673 ± 0.0221	0.4836 ± 0.0135	0.4382 ± 0.0114
RMSE/mm	0.8927 ± 0.0357	0.6194 ± 0.0186	0.6423 ± 0.0225	0.6582 ± 0.0257	0.5518 ± 0.0155	0.4858 ± 0.0126
MAX/mm	2.3152 ± 0.0926	1.4521 ± 0.0436	1.5327 ± 0.0536	1.3643 ± 0.0409	1.2985 ± 0.0389	1.2869 ± 0.0283

**Table 8:** Statistical significance tests of RMSE between M5 and the compared methods.

Comparison	Mean Diff. (mm)	*p*-Value	Significant?
M5 vs. BC	0.4069	<0.001	Yes
M5 vs. M1	0.1336	<0.001	Yes
M5 vs. M2	0.1565	<0.001	Yes
M5 vs. M3	0.1724	<0.001	Yes
M5 vs. M4	0.0660	0.003	Yes
M5 vs. LM-GA	0.0231	0.038	Yes
M5 vs. EKF+DQPSO	0.0320	0.032	Yes

**Table 9:** Tracking error statistics of polishing path compensation under different methods.

Metrics	BC	M1	M2	M3	M4	M5
MEAN/mm	0.847	0.623	0.598	0.612	0.512	0.438
RMSE/mm	0.963	0.708	0.731	0.746	0.586	0.498
MAX/mm	2.184	1.401	1.486	1.337	1.274	1.241
Points within ±0.5 mm (%)	12.5	34.8	38.2	36.5	52.3	68.5
Points within ±1.0 mm (%)	45.3	78.6	81.4	79.8	91.7	96.2

**Table 10:** Specific parameters of the polishing experiment.

Item	Detail
Workpiece	Φ150 mm flat ULE glass
Polishing tool	Φ30 polyurethane polishing disc
Polishing path	Based on the compensated paths of BC, M4 and M5
Polishing pressure (N)	15
Orbital speed (r/min)	80
Autorotation speed (r/min)	200
Polishing slurry	CeO_2_

## Data Availability

The data presented in this study are available on request from the corresponding author (the data are not publicly available due to privacy).
